# Methyl-CpG-binding 2 K271 lactylation-mediated M2 macrophage polarization inhibits atherosclerosis

**DOI:** 10.7150/thno.94738

**Published:** 2024-07-08

**Authors:** Liangqi Chen, Meiju Zhang, Xueyan Yang, Yanan Wang, Tuo Huang, Xin Li, Yunting Ban, Qifeng Li, Qingyuan Yang, Yongxiang Zhang, Yang Zheng, Di Wang, Xiaoqi Wang, Xiujie Shi, Maomao Zhang, Yong Sun, Jian Wu

**Affiliations:** 1 Department of Cardiology, The Second Affiliated Hospital of Harbin Medical University, Harbin, China.; 2 The Key Laboratory of Myocardial Ischemia, Chinese Ministry of Education, Harbin, China.; 3 Cardiac Rehabilitation Center, Department of Cardiology, The Second Affiliated Hospital of Harbin Medical University, Harbin, China.; 4 The Clinical Skills Center, The Second Affiliated Hospital of Harbin Medical University, Harbin, China.

**Keywords:** MeCP2 lactylation, H3K36me3, chromatin accessibility, macrophages polarization, atherosclerosis, exercise

## Abstract

**Rationale:** Posttranslational modifications of proteins have not been addressed in studies aimed at elucidating the cardioprotective effect of exercise in atherosclerotic cardiovascular disease (ASCVD). In this study, we reveal a novel mechanism by which exercise ameliorates atherosclerosis via lactylation.

**Methods:** Using ApoE^-/-^ mice in an exercise model, proteomics analysis was used to identify exercise-induced specific lactylation of MeCP2 at lysine 271 (K271). Mutation of the MeCP2 K271 lactylation site in aortic plaque macrophages was achieved by recombinant adenoviral transfection. Explore the molecular mechanisms by which motility drives MeCP2 K271 lactylation to improve plaque stability using ATAC-Seq, CUT &Tag and molecular biology. Validation of the potential target RUNX1 for exercise therapy using Ro5-3335 pharmacological inhibition.

**Results:** we showed that in ApoE^-/-^ mice, methyl-CpG-binding protein 2 (MeCP2) K271 lactylation was observed in aortic root plaque macrophages, promoting pro-repair M2 macrophage polarization, reducing the plaque area, shrinking necrotic cores, reducing plaque lipid deposition, and increasing collagen content. Adenoviral transfection, by introducing a mutant at lysine 271, overexpressed MeCP2 K271 lactylation, which enhanced exercise-induced M2 macrophage polarization and increased plaque stability. Mechanistically, the exercise-induced atheroprotective effect requires an interaction between MeCP2 K271 lactylation and H3K36me3, leading to increased chromatin accessibility and transcriptional repression of RUNX1. In addition, the pharmacological inhibition of the transcription factor RUNX1 exerts atheroprotective effects by promoting the polarization of plaque macrophages towards the pro-repair M2 phenotype.

**Conclusions:** These findings reveal a novel mechanism by which exercise ameliorates atherosclerosis via MeCP2 K271 lactylation-H3K36me3/RUNX1. Interventions that enhance MeCP2 K271 lactylation have been shown to increase pro-repair M2 macrophage infiltration, thereby promoting plaque stabilization and reducing the risk of atherosclerotic cardiovascular disease. We also established RUNX1 as a potential drug target for exercise therapy, thereby providing guidance for the discovery of new targets.

## Introduction

Atherosclerotic cardiovascular disease (ASCVD) is the leading cause of death worldwide 1. There is increasing evidence that exercise reduces ASCVD 2, 3. Lower risks of reinfarction, cardiac mortality, and all-cause mortality have been demonstrated in patients randomized to exercise treatment 4. These benefits are thought to be related to the modulation of epigenetics involved in coronary atherosclerosis 5. The role of exercise in epigenetics is being increasingly recognized 6, 7. Global DNA methylation in blood has been shown to be upregulated after exercise 8, 9. Regarding histone acetylation, early studies have addressed the implication of HDACs after exercise in altering expression of genes involved in metabolic processes through the transcription factor myocyte enhancer factor 2 10. Long-term exercise also induces DNA methylation of the Src homology 2 domain-containing transforming protein C1 in mononuclear cells, which promotes healthy microvascular aging and reduces the risk of cardiac disease 11. However, while the contribution of other epigenetic processes, such as DNA methylation and histone modifications, to exercise progression has been established, the role of posttranslational modifications (PTMs) in non-histones remains unclear. Moreover, it remains unknown how exercise induces the epigenetic changes that attenuate the progression of ASCVD.

PTMs are the covalent, enzymatic, or non-enzymatic attachments of specific chemical groups to amino acid side chains 12. They can modulate protein activity, localization, folding, and interactions with other biomolecules by altering the physicochemical properties of proteins 13. PTMs play a central role in remodeling chromatin structure or gene expression and collaborate with transcription factors and translational machinery in fine-tuning gene expression in disease states 14, 15. A previous study discovered a new PTM, lactylation, which is the addition of a lactyl (La) group to lysine residues in the tails of histone proteins 16. Lactate-derived histone lactylation is a recently discovered epigenetic modification that activates gene transcription 17. α-Myosin heavy chain undergoes lactylation at the Lys1897 residue to modulate the sarcomeric interaction between α-Myosin heavy chain and Titin, resulting in an alleviation of heart failure 18. Lactate is formed by the conversion of pyruvate via the enzyme lactate dehydrogenase with increasing exercise intensity 19, 20. An increase in arterial lactate concentration due to exercise appears to increase cardiac lactate uptake 21. In addition, excessive plaque accumulation leads to increased lactate production, and blood lactate may be higher in patients with established carotid atherosclerosis 22, 23. In this study, we hypothesized that the cardioprotective effect of exercise on ASCVD was related to lactylation modifications.

Methyl-CpG-binding protein 2 (MECP2), an X-linked gene (chromosome Xq28), encodes a nuclear protein with high affinity for methylated CpG islands in promoter regions, functioning as a transcriptional repressor or activator and regulator of chromatin structure 24, 25. MeCP2 recruits the negative regulators HDAC2 and Sin3A to form a repressor complex that promotes the deacetylation of histone tails, leading to gene silencing 26. The function of MeCP2 in neurodevelopmental disorders has been extensively studied. Phosphorylation of MeCP2 at serine 80 27, serine 421 28 and threonine 308 29 is required for neuronal activity. SUMOylation of MeCP2 at Lys 412 is required for transcriptional repression and synapse development 30. PTMs also critically function in MeCP2 regulation in cancer. One study reported that SIRT1 participates in the interaction of MeCP2 with ATRX and HDAC1 in breast cancer by modulating the acetylation of MeCP2 Lys171 31. MeCP2 is a vital epigenetic regulator of the macrophage response to stimuli and stressors 32. The role of MeCP2 in PTM has been intensively studied; however, whether MeCP2 regulates macrophage responses to exercise and contributes to atherosclerosis remains unclear.

In this study, we report that a better understanding of the regulation of macrophage polarization by exercise-driven lactylation of MeCP2 K271 will likely provide insights into pathways that could be exploited to potentially manipulate exercise towards an atheroprotective state. We break with traditional wisdom and find that exercise induces MeCP2 K271 lactylation and drives polarization of pro-repair M2 macrophages to promote stable plaque formation and ameliorate atherosclerosis, and overexpression of MeCP2 K271 lactylation reinforces this trend. Integrated CUT &Tag and ATAC-seq data showed that MeCP2 K271 lactylation directly recognizes and binds H3K36me3. This interaction enforces an optimal "repression" state of the downstream gene RUNX1, and mediates polarization of pro-repair M2 macrophages and increases the plaque stability, destroying the promotion effect of RUNX1 in ASCVD lesions. Overall, our study demonstrated that MeCP2 K271 lactylation-H3K36me3/RUNX1 in macrophages plays a critical role in the development of atherosclerosis.

## Methods

### Animals

ApoE deficient mice (ApoE^-/-^), backcrossed for ten generations on the C57BL/6J background, were obtained from Beijing Vital River Laboratory Animal Technology (Beijing, China). All animal experiments were performed with the approval of the Institutional Animal Care and Use Committee at the Second Affiliated Hospital of Harbin Medical University (sydwgzr2020-095) and in accordance with the Guidelines for the Care and Use of Laboratory Animals (Institute of Laboratory Animal Resources/National Institutes of Health, Bethesda, MD, USA).

All animals were maintained in rooms of the Second Affiliated Hospital of Harbin Medical University under specific pathogen-free (SPF) conditions. Housing rooms temperature was between 22-26 °C, with a relative humidity of 58% and a 12: 12-h light-dark cycle. Animals had ad libitum access to tap water and vacuum-packed pelleted feed. All male mice were fed with a high-fat diet (HFD) containing 21% fat, 20% protein and 50% carbohydrate (H10141, Beijing HFK Bioscience) at eight weeks of age. After 8 weeks, mice were randomly divided into control and experimental groups, and the specific exercise protocols were subsequently described. After completion of all protocols, mice were anaesthetized with 10% chloral hydrate, euthanized, and tissues were collected.

### Study population and exercise training protocol of participants

A total of 60 patients with stable coronary artery disease on treatment were selected for our study from the Second Affiliated Hospital of Harbin Medical University between December 2020 and June 2021. Inclusion criteria were: sinus rhythm, preserved left ventricular function (ejection fraction >50%), clinical stability for at least two weeks prior to entry to the study plus optimal and stable medical treatment. Exclusion criteria were: unstable angina, congestive heart failure, uncontrolled hypertension, valvular heart disease, impaired renal or hepatic function. Baseline characteristics of the study population are presented in supplemental Table 2. The exercise training protocol was developed following the recommendations of “The National Key R&D Program of China (Grant no. 2016YFC1301105)”. All study data have been uploaded to the China National Population and Health Science Data Platform. Patients in the exercise group were required to perform 30-60 min of moderate-intensity aerobic exercise 3-5 times/week for 12 weeks in addition to their usual care, while patients in the control group only received usual clinical care. Patients' peripheral blood will be collected at week 12 of the study for Western blot and qRT-PCR to detect changes in levels of MeCP2 K271 lactylation, H3K36me3 and RUNX1. During each session ECG, HR and BP were measured at baseline, at the end of each interval and at recovery. All participants or their families provided informed consent for inclusion before participation in the study, conforming to the Declaration of Helsinki. The current study was approved by the Ethics Committee of the Second Affiliated Hospital of Harbin Medical University, China (KY2020-156).

### Exercise training protocol of ApoE^-/-^ mice

To assess the effects of moderate-intensity aerobic treadmill training on mice models of atherosclerosis, we taught ApoE^-/-^ mice to perform 60-min exercise training on a motorized rodent treadmill with electric shock plate incentive (ZH-PT/5S, AN HUI, China) 5 days/week for 8 weeks. Exercise training started after 8 weeks of HFD. To allow acclimatization, the treadmill speed was initially set at 10 m/min for 10 min and increased to 18 m/min after a 2-min rest interval. All mice tolerated the exercise experiment well throughout the study. Except for exercise time in the exercise group, all mice were confined to their cages throughout the study.

### AAVs injection in ApoE^-/-^ mice

Recombinant adeno-associated virus (AAVs) vectors carrying Flag, Flag-tagged MeCP2 K271WT and MeCP2 K271R were purchased from Hanheng Biotechnology (Shanghai) Co. HFD-fed adult male ApoE^-/-^ mice for 8 weeks were randomly divided into 3 groups: the AAV-Flag group, the AAV-MeCP2 K271WT group, and the AAV-MeCP2 K271R group, and AAVs were injected into the tail vein in a single dose. Aortic tissues were taken by execution after 4 weeks for validation.

### Bone Marrow Derived Macrophages (BMDMs)

BMDMs were isolated from femurs and tibias of C57BL/6 mice by inserting a needle into the bone and flushing with DMEM. Macrophages were purified from cell suspensions by centrifugation (5 min, 1000 r, 4 °C). Cells were lysed with Red Blood Cell Lysing Buffer, centrifuged (8 min, 1500 r, 4 °C), and resuspended to remove red blood cells. Cells were grown at 37°C and 5% CO2 in Dulbecco's Modified Eagle Medium (DMEM) (Thermo Fisher Scientific) supplemented with 10% FBS (Biological Industries, Israel) and 1% penicillin/streptomycin. After the cells were seeded for 48 h in 6-well plates (1×10^6^ cells/mL), they were treated for 24 h with various doses of lactate (Sigma-Aldrich, S108838, 0, 5, 10, and 20 mM), 2-deoxy-D-glucose (2-DG) (MedChemExpress, HY-13966, 0, 1, 10, 20 mM), and sodium oxamate (Sigma-Aldrich, S123221, 0, 5, 10, 20 mM). All cell lines tested negative for mycoplasma.

### RNA silencing and Plasmids and Transfection of BMDMs

The siRNAs for siLDHA, siP300, siSETD2 and siHDAC3 were purchased from GenePharma (Shanghai, China). The Flag-tagged plasmids of AAV-MeCP2 K271 WT, AAV-MeCP2 K271R, and AAV-MeCP2 K271Q were obtained from Hanheng Biotechnology (Shanghai, China). According to the manufacturer's recommendations, siRNA and plasmids were transiently transfected using Lipofectamine 3000 (Invitrogen, Thermo Fisher Scientific, Waltham, MA, USA). Cell culture supernatants were collected at 24 h, and cell lysates were collected at 72 h.

### Mice/human peripheral blood mononuclear cells (PBMCs) isolation

Peripheral blood was collected and processed within 1 h. Plasma was collected after centrifugation at 4 °C (3000 rpm, 15 min). Monocytes were isolated from the cell fraction using the TBD Mouse PBMCs Isolation Kit (TBD science, TBD2011M) following the protocol described by the manufacturer.

### Analysis of ApoE^-/-^ mice aortic atherosclerosis

For macrophages marker analysis, F4/80^+^ macrophages were isolated from aortic root plaques using anti-F4/80 magnetic beads (Miltenyi Biotec, Germany) according to the manufacturer's instructions and subjected to analysis as described below. For macrophages marker analysis, F4/80^+^ macrophages were isolated from aortic root plaques using anti-F4/80 magnetic beads (Miltenyi Biotec, Germany) according to the manufacturer's instructions and subjected to analysis as described below. To assess the morphology of the atherosclerotic plaques and to visualize the lipid content in the atherosclerotic plaques, and analyze collagen content, fibrous cap thickness and necrotic core formation. We performed HE staining, Oil red O staining and Masson trichrome staining. Heart samples were fixed in 4% paraformaldehyde, dehydrated in 30% sucrose solution, immersed in OCT (Tissue-Tek, Sakura, Torrance, CA), and serial cryosections (7 μm) were performed. For HE staining, slides were stained in hematoxylin for 3 min and counterstained with eosin for 8 min. For Oil red O staining, aortic root slides were stained with Oil red O for 15 min and counterstained in hematoxylin for 3 min. The HE staining kit, Oil red O staining kit and Masson trichrome staining kit were obtained from Solarbio (Beijing, China). Images were photographed with an Olympus BX41 microscope (Tokyo, Japan), and plaque quantification was evaluated with Image-Pro Plus 6.0 software.

### Immunofluorescence staining and confocal microscopy

For immunofluorescence microscopy of tissue, cryosections were prepared as previously described. The 7-μm sections placed on glass slides were fixed with 100% cold acetone for 15 min and permeabilized with 0.1% Triton X-100 (Biosharp, Hefei, China). After blocking in 1% bovine serum albumin (BSA), slides were incubated with primary antibodies overnight at 4 °C. Slides were then incubated with the fluorescently labeled secondary antibodies (abcam, ab150079, ab150077, ab150131, ab150157, ab150116, ab150129) at 37 °C for 2 h in the absence of light, followed by 4',6-diamidino-2-phenylindole (DAPI) (Beyotime, C1005) to counterstain the nuclei.

For cell immunofluorescence microscopy, cells grown on 24-well chamber slides were washed three times in phosphate-buffered saline (PBS) for 5 min and fixed in 4% formaldehyde for 5 min. Cells were then blocked with 1% BSA for 20 min at room temperature and permeabilized with 0.1% Triton X-100 in PBS. After blocking with 1% BSA, cells were incubated with primary antibodies overnight at 4 °C. Cells were washed three times in PBS and incubated with fluorescently labeled secondary antibodies (abcam, ab150079, ab150077, ab150131, ab150157, ab150116) for 1 h at 37 °C. Unbound secondary antibodies were removed by washing three times with PBS, and cells were then stained with DAPI for 5 min at room temperature. After washing three times in PBS, coverslips were mounted onto glass slides. Sections were visualized and photographed using a confocal laser microscope (Zeiss LSM 800). The used primary antibodies include anti-Pan Kla (PTMab, PTM-1401RM, 1: 50), anti-F4/80 (Abcam, ab16911, 1: 50), anti-CD206 (RD SYSTEMS, AF2535, 1:50), anti-CD86 (NOVUSBIO, BP2-25208, 1:50), anti-MeCP2K271la (PTM BIO Inc, CL040801, 1:50), anti-H3K36me3 (PTMab, PTM-625RM, 1:50) and anti-RUNX1 (abcam, ab92336, 1:50) antibodies.

### Western blot analysis

Routine procedures were followed for protein extraction. Protein extracts were separated by 8-15% SDS-polyacrylamide gel electrophoresis (SDS-PAGE) and subsequently transferred to 0.22 μm polyvinylidene fluoride (PVDF) membrane (Merck Millipore, Billerica, MA, USA). Membranes were blocked with 5% nonfat milk for 2 h at room temperature and then incubated with primary antibodies overnight at 4 °C. After washing three times in TBS-t, the membranes were incubated with horseradish peroxidase-conjugated secondary antibodies at room temperature for 1 h. Immunoblots were visualised using a Tanon 5100 system (Tanon, Shanghai, China), and densitometry analyses were performed using Image J software. Primary antibodies included: anti-Pan Kla (PTMab, PTM-1401RM, 1:1000), anti-MeCP2 (abcam, ab53197, 1:1000), anti-MeCP2K271la (PTM BIO Inc, CL040801, 1:1000), anti-Histone H3 (proteintech, 1768-1-AP, 1:1000), anti-H3K36me3 (PTMab, PTM-625RM, 1:4000), anti-H3K36me1 (PTMab, PTM-623RM, 1:1000), anti-H3K36me2 (PTMab, PTM-662RM, 1:1000), anti-H3K27me3 (PTMab, PTM-616, 1:3000), anti-H3K9me3 (PTMab, PTM-5002, 1:3000), anti-RUNX1 (abcam, ab92336, 1:50), anti-P300 (Cell Signaling TECHNOLOGY, 67621S, 1:1000), anti-HDAC3 (proteintech, 10255-1-AP, 1:1000), anti-SETD2 (proteintech, 11231-1-AP, 1:1000), anti-β-actin (ZSGB-BIO, TA -09, 1:1000), anti-normal rabbit IgG (Cell Signaling TECHNOLOGY, 2729S, 1:1000), anti-IL-10 (proteintech, 60269-1-Ig, 1:1000), anti-TNF Alpha (proteintech, 17590-1-AP, 1:1000), anti-Arginase-1 (proteintech, 16001-1-AP, 1:1000), anti-iNOS (abcam, ab178945, 1:1000). Horseradish peroxidase conjugated secondary antibodies included horseradish peroxidase conjugated goat anti-rabbit IgG (ZSGB-BIO, 1:10000), horseradish peroxidase conjugated goat anti-rabbit IgG (abcam, ab6721, 1:3000), horseradish peroxidase conjugated goat anti-mouse IgG (ZSGB-BIO, ZB-2305, 1:10000).

### Co-Immunoprecipitation (Co-IP)

Cells were lysed in NP-40 immunoprecipitation lysis buffer supplemented with 5% PMSF (Beyotime, Shanghai, China) for 30 min on ice. After clarification by centrifugation, the total protein lysate were immunoprecipitated with anti-P300 (Cell Signaling TECHNOLOGY, 67621S), anti-MeCP2K271la (PTM BIO Inc, CL040801), anti-H3K36me3 (PTMab, PTM-625RM), anti-HDAC3 (proteintech, 10255-1-AP) and anti-FLAG (DYKDDDDK tag Rabbit Polyclonal antibody) antibodies overnight at 4 °C. Protein A/G magnetic beads for IP (bimake, B23202) were then washed three times with NP-40 lysis buffer and then incubated with the immune complexes for 4 h at 4 °C. The clear supernatant was transferred into a new tube as input. After mixing with sample buffer, the immune complexes were boiled at 100 °C for 10 min and detected by immunoblots.

### ChIP-qPCR

Cells were harvested and cross-linked with 4% formaldehyde for 10 min at room temperature followed by quenching with 125 mM glycine for 5 min. Washed cells were resuspended in lysis buffer (1% SDS, 5 mM EDTA, and 50 mM Tris-HCl, pH 8.1) supplemented with protease inhibitors and then sonicated on ice for 60 s to shear cross-linked DNA to a length of approximately 300 bps. For immunoprecipitation, sheared chromatin was incubated with anti-MeCP2K271la (PTM BIO Inc, CL040801), anti-H3K36me3 (PTMab, PTM-625RM), and anti-Normal Rabbit IgG (Cell Signaling TECHNOLOGY, 2729S) antibodies overnight at 4 °C, and chromatin immune complexes were conjugated to protein A/G Sepharose beads (MCE) for an additional 2 h. The beads were sequentially added once to TSE I buffer (0.1% SDS, 1% Triton X-100, 2 mM EDTA, 150 mM NaCl, and 20 mM Tris-HCl, pH 8.0); TSE II buffer (0.1% SDS, 1% Triton X-100, 2 mM EDTA, 500 mM NaCl, and 20 mM Tris-HCl, pH 8.0); TSE III buffer (0.25 M LiCl, 1% Nonidet P-40, 1% sodium deoxycholate, 1 mM EDTA, and 10 mM Tris-HCl, pH 8.0). The immunoprecipitated complex was washed and de-cross-linked at 55°C for 12 h in wash buffer (1% SDS and 0.1 M NaHCO3). ChIP assays were evaluated using the SimpleChIP Enzymatic Chromatin IP Kit (Cell Signaling Technology, Danvers, MA, USA). The primers used were listed in Table S1.

### RNA purification and quantitative reverse transcriptase PCR (qRT-PCR)

Total RNA was extracted from cells using TRIzol reagent (Invitrogen, Carlsbad, USA), and purity was determined using a spectrophotometer (BioSpec-nano). For mRNA detection, first strand cDNA was synthesized from purified total RNA using the Transcriptor First Strand cDNA Synthesis Kit (Roche, Basel, Switzerland) reverse transcription protocol. Then, quantitative real-time PCR was performed using a Transcriptor First Strand cDNA Synthesis Kit (Roche, Basel, Switzerland) for 40 cycles at 95 °C for 10 s, 60 °C for 30 s, and 72 °C for 30 s. The primer sequences used are listed in Table S1. qRT-PCR was repeated for each gene in at least three separate experiments on three separate occasions.

### Assay for Transposase Accessible Chromatin (ATAC)-sequencing and analysis

For the nuclei preparations, PBMCs were collected from mice, washed three times in cold PBS, and lysed for 10 min at 4 °C in a rotary mixer. After microscopic inspection of the purity and integrity of the nuclei, the nuclear pellets were labeled with Tn5 transposase (TruePrep DNA Library Prep Kit V2 for Illumina) for 30 min at 37 °C and purified with AMPure DNA magnetic beads. During tagmentation, a mild detergent was chosen to keep the nuclei intactness. The resulting library fragments were run on an Agilent Tapestation 2200 (Agilent Technologies) using a D5000 DNA ScreenTape and finally sequenced on the Illumina HiSeq X Ten platform (San Diego, CA, United States) in 150 PE mode. Biological replicates were processed in duplicate per biological sample.

Prior to mapping, cutadapt (1.9.1) was performed to standard quality control steps of next-generation sequencing. Alignment to the reference genome was performed using Bowtie 2.2.6, reads assigned to the mitochondrial genome were removed using removeChrom (https://github.com/jsh58/harvard), and PCR duplicates were removed with Picard (version 1.126). ATAC-seq data were evaluated using the Transcription Start Site (TSS) enrichment score, and the normalized signal value in the middle of the distribution was considered our TSS enrichment metric. The potential differentially accessible regions (DAR) and Gene Ontology (GO) enrichment detection were performed using the R package. Motif analyses on DARs were performed using the MEME suite, and all sequencing traces were displayed using Integrated Genomic Viewer (IGV 2.3.61).

### Cleavage Under Targets and Tagmentation assay (CUT &Tag)

CUT &Tag was performed according to the manufacturer's instructions. Briefly, PBMCs from mice were collected and washed twice in ice-cold PBS. After centrifugation at 1,600 rpm for 5 min at 4 °C, aliquots of cells (1×10^5^) were washed twice in wash buffer by gentle pipetting and bound to activated concanavalin A-coated magnetic beads (Bangs Laboratories) for 15 min at room temperature. The unbound supernatant was removed and bead-bound cells were resuspended in antibody buffer (20 mM HEPES pH 7.5, 150 mM NaCl, 0.5 mM spermidine, 0.05% digitonin (Sigma-Aldrich), 2 mM EDTA (Beyotime, C0201), 0.1% BSA, 1×protease inhibitor cocktail). Subsequently, the cells were incubated with specific primary antibodies overnight at 4 °C. The primary antibody was removed and cells were incubated with the appropriate secondary antibodies supplemented with Dig-Wash buffer (20 mM HEPES pH 7.5, 150 mM NaCl, 0.5 mM spermidine, 0.05% digitonin, 1×protease inhibitor cocktail) for 1 h at room temperature. The hyperactive pA-Tn5 transposase adaptor complex (1:200) was prepared with Dig-med buffer (0.05% digitonin, 20 mM HEPES, pH 7.5, 300 mM NaCl, 0.5 mM spermidine, 1× protease inhibitor cocktail). Cells were incubated with pA-Tn5 for 1 h at room temperature and then washed three times in Dig-med buffer. Next, cells were resuspended in Tagmentation buffer (10 mM MgCl2 in Dig-300 buffer) and incubated for 1 h at 37 °C to activate the transposome. After tagmentation was completed, DNA was isolated, amplified, and purified to enrich fragment libraries. Libraries were quantified using the VAHTS Library Quantification Kit and sequenced on Illumina NovaSeq.

### Liquid Chromatography-mass Spectrometry (LC-MS/MS) analysis

The extracted protein solutions were digested overnight with trypsin at a 1:50 trypsin-to-protein mass ratio. Tryptic peptides were dissolved in IP buffer (100 mmol/L NaCl, 1 mmol/L EDTA, 50 mmol/L Tris-HCl, 0.5% NP-40, pH 8.0). Supernatants were collected and transferred to prewashed anti-lactylation antibody beads (PTM Bio, PTM-1404), which were stored overnight with gentle shaking at 4 °C. The bound peptides were washed from the beads three times with 0.1% trifluoroacetic acid. Subsequently, the eluted solutions were collected and the peptides were desalted and vacuum dried according to the manufacturer's protocols. For LC-MS/MS analysis, peptides were dissolved in solvent A (97% water, 3% acetonitrile, and 0.1% formic acid) and analyzed using a NanoElute ultraperformance liquid chromatography (UPLC) system running at 450 nL/min in mobile phases. The gradient began with an increase from 6% to 22% solvent B (100% acetonitrile, 0.1% formic acid) over 44 min, 23 to 30% in 10 min, and increased to 80% in 3 min, followed by equilibration at 80% in the last 3 min. Then the eluted peptides were ionized by the capillary ionization method and introduced into the timsTOF Pro mass spectrometer for MS /MS. The m/z (mass-to-charge ratio) scan range of a full MS scan was 400 to 1500 with 30 s dynamic exclusion. GO analyses were performed using eggNOG-mapper (v2.0) based on the eggNOG database. Protein structure annotations were based on the Pfam database and the PfamScan tool.

### Flow cytometric analysis

BMDMs were treated with lactate and Ro5-3335 and collected as previously described. To obtain arterial cells, mice were deeply anesthetized and hearts were extensively flushed with PBS to remove peripheral blood cells. Hearts and arteries were dissected, minced with fine scissors, and digested in collagenase I (450 U/ml), collagenase XI (125 U/ml), DNase I (60 U/ml), and hyaluronidase (60 U/ml) (Sigma-Aldrich) at 37 °C for 1 h. Then, cells were collected by homogenization through a 40-μm nylon mesh and lysed with red blood cell lysis buffer (BioLegend). For isolated aortic root plaque macrophages as described above. Collected cells were stained with PE anti-mouse CD86 (BioLegend, 105007), APC anti-mouse F4/80 (BioLegend, 123115), PerCP/cyanine5.5 anti-mouse/human CD11b (BioLegend, 117307) and FITC anti-mouse CD206 (MMR) (BioLegend, 141703) antibodies for 30 min at 4 °C away from light. Subsequently, stained cells were washed, fixed, and permeabilized in Fix/Perm buffer (Invitrogen, Thermo Fisher Scientific, Waltham, MA, USA). Flow cytometry was evaluated with FACSDiva version 6.1.3 (BD Biosciences, San Jose, CA, USA), and analyses were performed with FlowJo_V10.6.2 (TreeStar, Ashland, OR, USA).

### Serum lactate, triglycerides, HDL cholesterol, cholesterol, glucose determination

At the end of the training, blood samples were collected and centrifuged at 3000 rpm for 15 min to obtain serum. Lactate concentrations in mouse serum samples were determined using indicated assay kits (Sigma-Aldrich, MAK064) according to the manufacturer's instructions.

### Molecular docking of MeCP2 K271 lactylation to H3K36me3

Prediction of molecular docking of MeCP2 K271 lactylation to H3K36me3 was performed using AutoDock 4.0 software. The proteins obtained were processed to retain the original charge and add hydrogen atoms. The corresponding crystal structures were retrieved from the PDB database. Interaction analyses were performed using Discoverystudio and Ligplot.

### Ro5-3335 injection

All experiments were performed after animals were treated with HFD for 8 weeks. ApoE^-/-^ mice (both sexes with approximately equal M/F distribution) were treated with an intraperitoneal injection of Ro5-3335 (MedChemExpress, HY-108470, 25 mg/kg body weight) dissolved in DMSO. The same volume of DMSO was administered to the mice in the control group. The injections were performed twice a week for 4 weeks. The mice were weighed each week to adjust the dose.

### Statistical analysis

All non-sequencing data statistical analysis was performed in GraphPad Prism version 9.1.0. The numbers in figure legends indicated biological replicates performed in each experiment. When only two groups were compared, an unpaired two-tailed Student' t test was used. Comparisons between multiple groups were calculated by one-way Fisher's LSD post hoc test or two-way ANOVA analysis. Data were reported as mean ± SDs. 0.05 was considered statistically significant.

## Results

### Exercise induced pro-reparative M2 macrophage polarization and created a demethylated environment

**Figure 1:**
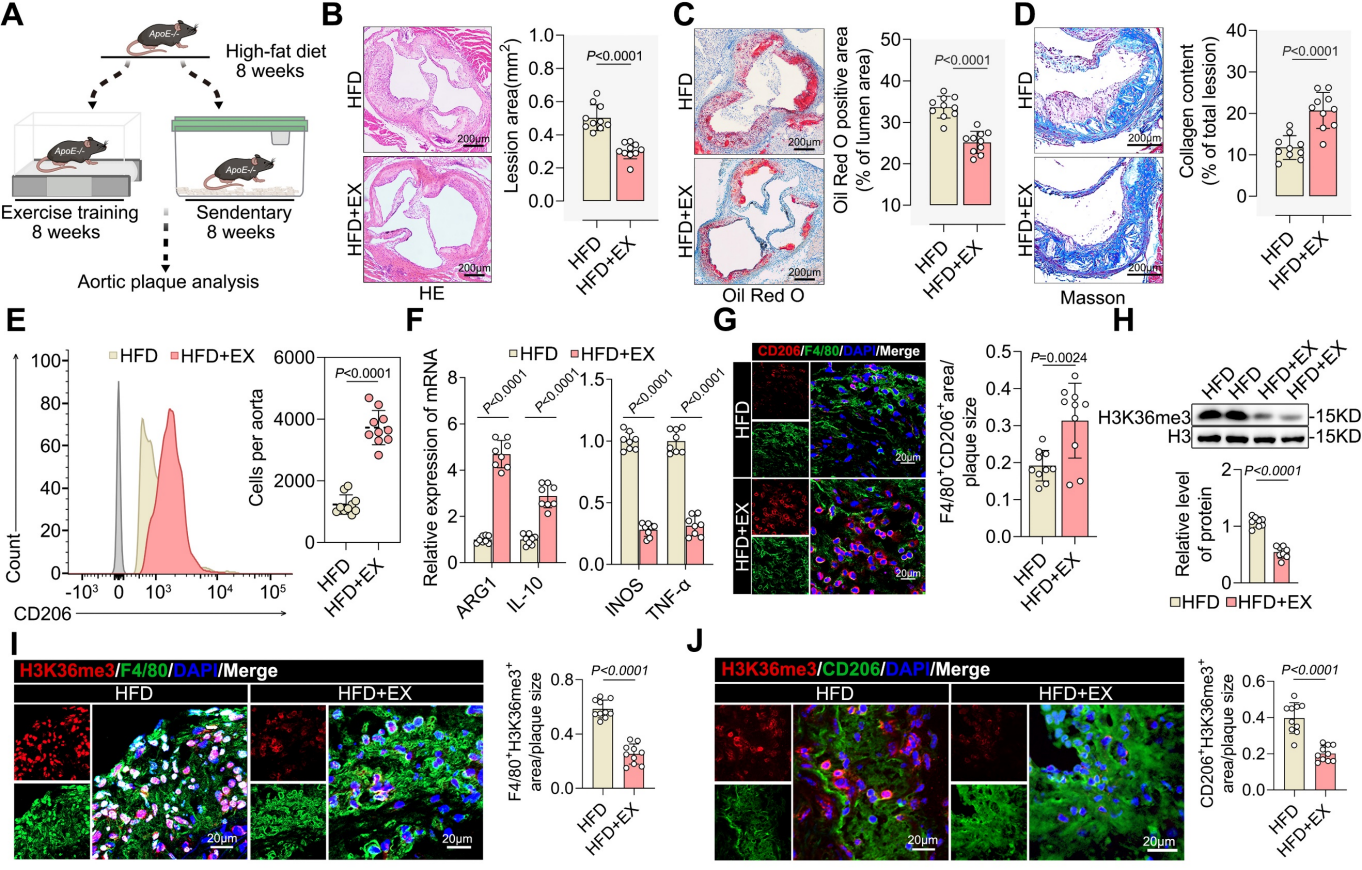
** Exercise induced pro-reparative M2 macrophages polarization and created a demethylated environment. A.** Schematic representation of the ApoE^-/-^ mice exercise model. **B-D.** Representative images and quantification of atherosclerotic area in cross-sections of aortic root of ApoE^-/-^ mice by HE **(B)**, Oil Red O staining **(C)** and Masson **(D)**. Scale 200 μm; 10 mice per group. **E.** Representative flow cytometric graph and quantification of CD206 macrophages in isolated aortic root plaque macrophages of ApoE^-/-^ mice. 10 mice per group. **F.** qRT-PCR analysis of M2-like genes (left) and M1-like genes (right) in isolated aortic root plaque macrophages. 8 mice per group. **G.** Representative immunofluorescence co-staining images for macrophage maker F4/80 and M2 macrophage maker CD206 in aortic root plaques of ApoE^-/-^ mice, with quantification of CD206 intensity. 10 mice per group. Scale 20 µm. n represents number of independent samples. **H.** Western blot analysis of protein expression level of H3K36me3 in isolated aortic root plaque macrophages of ApoE^-/-^ mice, with quantification of proteins. 8 mice per group. **I, J.** Representative images of immunofluorescence co-staining for H3K36me3 together with macrophage maker F4/80 **(I)** and M2 macrophage maker CD206 **(J)** in aortic root plaques from ApoE^-/-^ mice, with quantification of H3K36me3 intensity. 10 mice per group. Scale 20 µm. All data are shown as mean ± SD. Data were analyzed by two-tailed Student's t-test.

To clarify the atheroprotective effect of exercise, we established a model of exercise (HFD+EX) or sedentary (HFD) for eight weeks in HFD-fed ApoE^-/-^ mice (Figure 1A). As expected, exercise significantly decreased plaque area (Figure 1B) and lipid infiltration (Figure 1C), whereas collagen deposition increased (Figure 1D) in the aortic root of ApoE^-/-^ mice. We also found that exercise lowered the levels of the atherosclerosis risk factors serum triglycerides, serum cholesterol, serum glucose, and body weight while significantly elevating the level of the atherosclerosis protective factor serum HDL (Figure S1A-S1E). Furthermore, exercise increased the proportion of pro-repair M2 macrophages in the aortic root plaques (Figure 1E). We found an increase in M2-like genes ARG1 and IL-10 and a decrease in M1-like genes INOS and TNF-α (Figure 1F). Immunofluorescence also showed that exercise boosted pro-repair M2 macrophage infiltration into the aortic root plaques (Figure 1G). Surprisingly, exercise created an environment that facilitated histone H3K36me3 demethylation in the isolated aortic root plaque macrophages (Figure 1H). Furthermore, the level of H3K36me3 was lower in PBMCs from exercise-trained patients compared with that in the control samples (Figure S1F). We found that the proportion of macrophage-associated nuclei that stained positive for H3K36me3 was lower than that in the plaques of the HFD+EX group (Figure 1I). Co-staining of plaques for H3K36me3 and the M2 macrophage marker CD206 showed a onefold decrease in the HFD+EX group compared with that in the sedentary group (Figure 1J). These results indicate that H3K36me3 may be involved in the regulation of exercise-induced repair of M2 macrophage polarization. Collectively, exercise induces pro-reparative M2 macrophage polarization in plaques and inhibits the progression of atherosclerosis. Additionally, the demethylation of H3K36me3 may contribute to this process.

### Exercise drove lactylation to demethylate H3K36me3 and promote pro-repair M2 macrophage polarization in ApoE^-/-^ mice

**Figure 2:**
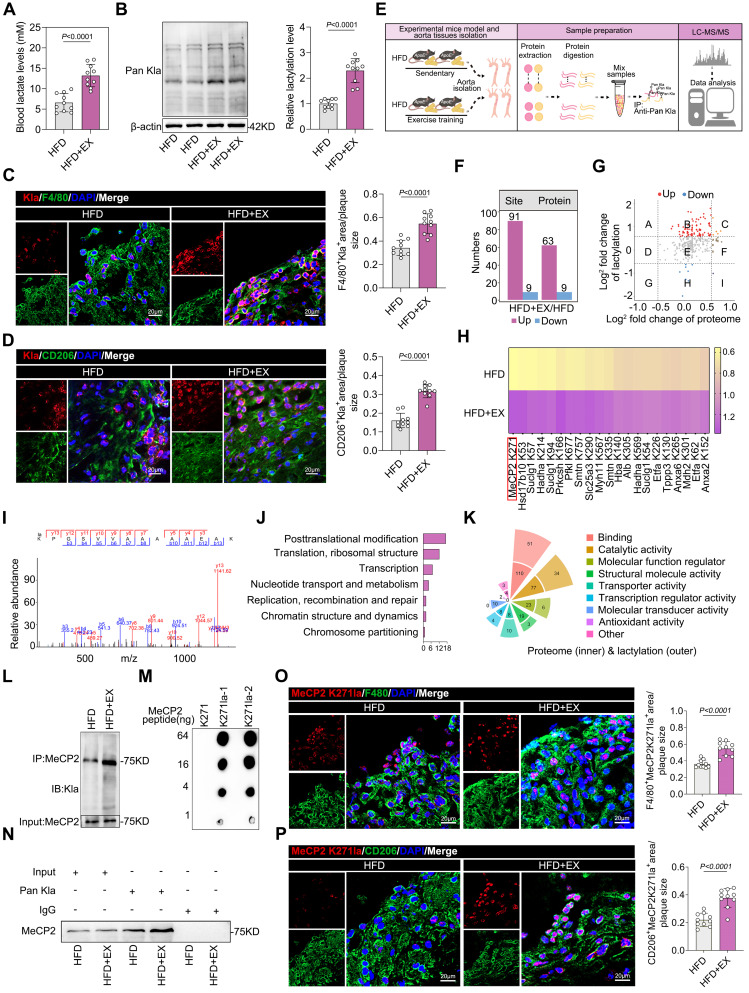
** Identification of MeCP2 K271 lactylation in macrophages from ApoE^-/-^ mice in response to exercise. A.** Lactate level trends in serum of exercise and sedentary treated ApoE^-/-^ mice. 10 mice per group. **B.** Western blot analysis of Pan Kla levels in isolated aortic root plaque macrophages of ApoE^-/-^ mice, with quantification of protein levels. 10 mice per group. **C, D.** Representative immunofluorescence co-staining images for Pan Kla together with macrophage maker F4/80 **(C)** and M2 macrophage maker CD206 **(D)** in aortic root plaques of ApoE^-/-^ mice, with quantification of Pan Kla intensity. 10 mice per group. Scale 20 µm. **E.** Schematic representation of the experimental workflow for LC-MS/MS of Kla in aorta of of sedentary and exercise trained ApoE^-/-^ mice. **F.** Numbers of differentially expressed lactylation proteins (DELPs) after exercise. **G.** Scatter plot showing the relationship between proteins exhibiting remarkable Kla changes and proteins exhibiting remarkable changes in levels. **H.** Heatmap showing the top 21 DELPs with increased and decreased lactylation after exercise. **I.** Mass spectrum of MeCP2 peptide showing K271 lactylation sites in ApoE^-/-^ mice. **J.** Gene Ontology terms of representative function proteins exhibiting remarkable Kla changes or remarkable changes in levels. **K.** Molecular functional distribution of proteins exhibiting remarkable Kla changes or remarkable changes in levels. **L.** Immunoprecipitations were performed in isolated aortic root plaque macrophages of ApoE^-/-^ mice with anti-MeCP2 antibody followed by Western blot with specific anti-Pan Kla antibody. **M.** The specificity of antibodies against MeCP2 K271 lactylation was verified by dot blot assays. PVDF membrane was spotted with the indicated amounts of non-lactylated or lactylated MeCP2 peptides and immunoblotted with the indicated antibodies. **N.** Immunoprecipitations were performed in isolated aortic root plaque macrophages of ApoE^-/-^ mice with anti-Pan Kla or anti-IgG antibody followed by Western blot with specific anti-MeCP2 antibody. **O, P.** Representative immunofluorescence co-staining images for anti-MeCP2 K271la antibody together with macrophage maker F4/80 **(O)** and M2 macrophage maker CD206 **(P)** in aortic root plaques of ApoE^-/-^ mice, with quantification of MeCP2 K271 lactylation intensity. 10 mice per group. Scale 20µm. All data are shown as mean ± SD. Data were analyzed by two-tailed Student's t-test.

This resulted in a significant increase in the arterial lactate concentration. Colorimetric assays showed that lactate concentration was approximately twofold higher in the HFD+EX group than in the HFD group (Figure 2A). Because lactate functions as a precursor of a metabolic substrate that stimulates lactylation, it is reasonable to hypothesize that pan-lysine lactylation (pan-Kla) may be altered in the HFD+EX group. Western blot analysis showed that exercise increased Pan Kla levels in the aortic root plaques (Figure 2B). Treatment of BMDMs with lactate and the glycolysis inhibitor 2-DG, as well as oxamate, validated lactate-driven Pan-Kla (Figure S2A-S2C). Immunofluorescence co-staining of Pan Kla with the macrophage markers F4/80 and CD206 revealed that exercise elevated the levels of Pan Kla in macrophages (Figure 2C), especially in pro-repair M2 macrophages (Figure 2D) in the aortic root plaque. Similarly, our in vitro experiments further confirmed our findings that lactate stimulation promoted M2-like gene expression and pro-repair M2 macrophage polarization and that inhibition of lactate production suppressed this effect (Figure S2D-S2I). Furthermore, we found that lactate treatment mediated the demethylation of H3K36me3 and decreased SETD2 (Figure S2J-S2K), which is thought to catalyze H3K36 methylation 33, 34. Thus, it is reasonable to conjecture that exercise may drive lactylation to demethylate H3K36me3 and promote the pro-repair of M2 macrophage polarization.

### Proteome-wide analysis of ApoE^-/-^ mice identified the lactylation of MeCP2 K271 as a rewarding target

To obtain a comprehensive overview of exercise-mediated lactylation, we collected aortas from HFD and HFD+EX animals and performed LC-MS/MS analysis to assess protein lactylation modifications of lysine residues (Figure 2E). Compared with that in the HFD group, only an average Kla enrichment or log2 fold change of protein above 1.5 or below -1 was considered a remarkable change and analyzed further. Under exercise conditions as differentially expressed lactylated proteins (DELPs), we identified 91 sites of 63 proteins with upregulated lactylation and nine sites of nine proteins with downregulated lactylation (Figure 2F). The scatter plot shows that 25% of the proteins that exhibited Kla expression were upregulated after exercise in ApoE^-/-^ mice (log2 fold change>1.5), with no changes in protein levels (gray dots) (Figure 2G). Heatmap analysis showed the relative quantification of the top 20 proteins with upregulated or downregulated lactylation (Figure 2H). MeCP2 K271 substrate site presented the greatest change. Mass spectrometry analysis also revealed that MeCP2 contains a single lactylation site at K271 (Figure 2I), suggesting that this may be a critical lactylation site. Gene Ontology analysis of the identified DELPs performed to characterize the function of changed lactylated proteins indicated that DELPs mostly serve functions in posttranslational modification and binding (Figure 2J and 2K). Potential differential analysis of Kla-regulated intracellular signaling pathways revealed that those exhibiting altered levels were largely associated with signal transduction mechanisms and transcription (Figure S3B). The protein-protein interaction network of DELPs using CytoScape software also validated the critical role of MeCP2 in exercise-mediated lactylation to improve atherosclerosis (Figure S3E). Moreover, the level of MeCP2 K271 lactylation was significantly higher in the exercise population than in the controls (Figure S3F). Therefore, we focused on MeCP2 to investigate the biological consequences of exercise-modulated lactylation. We performed co-IP to identify MeCP2 lactylation sites in aortic root plaque macrophages isolated from ApoE-/- mice. Immunoprecipitation was performed using a highly specific antibody against MeCP2, followed by immunoblotting with an anti-pan-Kla antibody (Figure 2L). Immunoprecipitation with an anti-pan Kla antibody was performed in the same manner, followed by immunoblotting with a MeCP2 antibody (Figure 2N). The results showed that exercise promoted MeCP2 lactylation. To determine whether the MeCP2 K271 site is lactylated in vivo, we developed antibodies against lactylated MeCP2 K271. The specificity of these antibodies was verified by dot blot assays with the corresponding peptides with or without Kla modification (Figure 2M). As expected, immunofluorescence co-staining of the MeCP2 K271la antibody with F4/80 (Figure 2O) and CD206 (Figure 2P) revealed a remarkable increase in MeCP2 K271 lactylation in the HFD+EX group. Collectively, these observations support the notion that lactylation of the MeCP2 K271 site is a key modification in exercise-mediated atheroprotective effects.

### MeCP2 K271 was lactylated by p300 and delactylated by HDAC3 in vitro

To further explore lactate-induced lactylation sites in macrophages, we mutated MeCP2 K271 to arginine (R) and glutamine (Q) (K271R and K271Q) to mimic the delactylation state of the proteins. As shown in Figure S4A and S4B, both K271R and K271Q point mutations transfected with FLAG-tagged adeno-associated virus (AAV) resulted in lower lactylation compared with that of MeCP2 K271 wild type (WT). To test whether MeCP2 K271 is the key lactylation site for lactate stimulation, the cells were treated with a lactate concentration gradient. Western blot and immunofluorescence analyses showed increased MeCP2 K271 lactylation in response to lactate treatment (Figure S4C and S4E). However, lactate treatment had no significant effect on the lactylation of MeCP2 K271R and K271Q mutants (Figure S4D). These data confirm that K271 is the major lactylation site of MeCP2 and that lactylation can be induced by exogenous lactate. These data suggest that the acetyltransferase P300 can transfer lactate-transformed L-lactyl-CoA to protein lysine residues 16. We transfected with siP300, whole-cell lysates were prepared, and western blotting was performed with the indicated antibodies. Knockdown of P300 significantly reduced MeCP2 K271 lactylation, which could be reversed by exogenous lactate (Figure S4G). Thus, we conclude that P300 is a transferase for MeCP2 K271 lactylation. Moreno-Yruela et al. demonstrated that HDAC3 is the most effective scavenger of L- and d-lactate lysine in vitro 35. Therefore, we investigated whether HDAC3 modulates the delactylation of MeCP2 K271. To clarify this, we transfected cells with HDAC3 and performed western blotting with an anti-MeCP2 K271la antibody, which showed that HDAC3 delactylated MeCP2 K271 in a dose-dependent manner (Figure S4J). Moreover, HDAC3 knockdown significantly increased the lactylation of MeCP2 K271, which was enhanced by exogenous lactate treatment (Figure S4I). Immunoprecipitation also confirmed the interaction of HDAC3 with the lactylated MeCP2 K271 in the nuclei (Figure S3K). Thus, our experiments demonstrated that exogenous lactate can regulate the lactylation of MeCP2 K271 and can be modulated by P300 and HDAC3 in BMDMs. In conclusion, our study demonstrated that the lactylation of MeCP2 K271 can be catalyzed by P300 and de-lactylated by HDAC3.

### The lactylation of MeCP2 K271 promoted the regression of atherosclerosis and increased the accumulation of pro-reparative M2 macrophages

**Figure 3:**
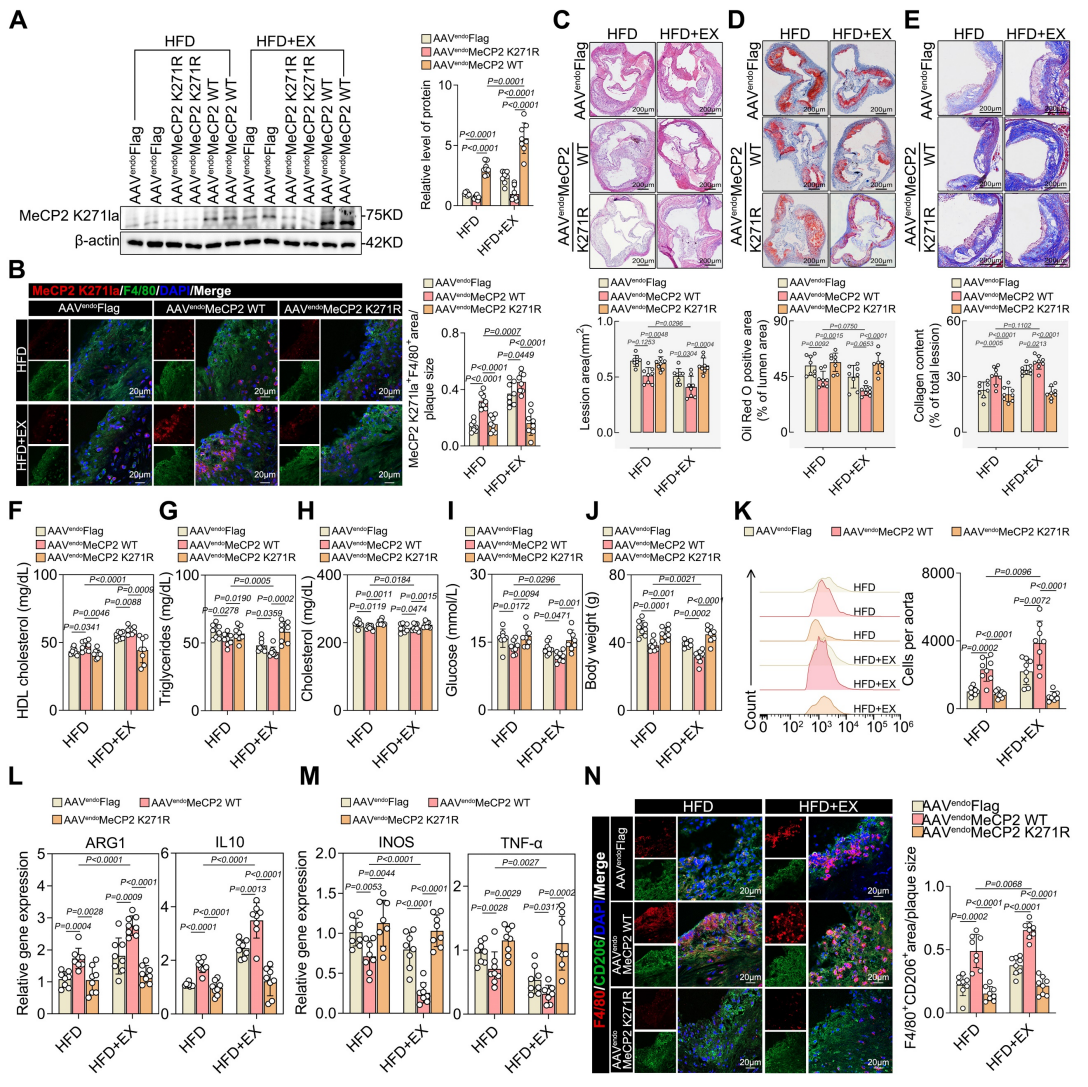
** Overexpression of MeCP2 K271 attenuated atherosclerosis and mediated M2 macrophage polarization in ApoE^-/-^ mice. A.** Western blot analysis of MeCP2 K271 lactylation levels in isolated aortic root plaque macrophages, with quantification of protein levels. 8 mice per group. **B.** Representative immunofluorescence co-staining images with anti-Pan Kla antibody and macrophage maker F4/80 in aortic root plaques, with quantification of Pan Kla intensity. 10 mice per group. Scale 20 µm. **C-E.** Representative images and quantification of atherosclerotic area of HE **(C)**, Oil Red O staining **(D)** and Masson **(E)** staining in cross-sections of aortic root. Scale 200 μm; 8 mice per group. **F-J.** Measurement of atherosclerosis protective/risk factor levels: serum HDL cholesterol **(F)**, serum triglycerides **(G)**, serum cholesterol **(H)**, serum glucose **(I)** and body weight** (J)**. 8 mice per group. **K.** Representative flow cytometric graph and quantification of CD206 macrophages in isolated aortic root plaque macrophages. 8 mice per group. **L, M.** qRT-PCR analysis of M2-like genes **(L)** and M1-like genes **(M)** in isolated aortic root plaque macrophages. 8 mice per group. **N.** Representative immunofluorescence co-staining images with M2 macrophage marker CD206 and macrophage maker F4/80, with quantification of CD206 intensity. 10 mice per group. Scale 20 µm. All data are shown as mean ± SD. Data were analyzed by one-way ANOVA with Fisher's LSD post hoc test.

To further assess the physiological effects of MeCP2 K271 lactylation, we generated recombinant AAV vectors packaged with FLAG-tagged MeCP2 K271WT and MeCP2 K271R. Eight weeks after the HFD diet, adult male ApoE^-/-^ mice were injected intravenously with 1×10^12^ AAV viral particles and sacrificed four weeks later. Western blotting revealed that AAV-MeCP2 K271R stimulation markedly downregulated MeCP2 K271 lactylation, which was not reduced by exercise (Figure 3A). Immunofluorescence revealed that AAV-MeCP2 K271WT treatment decreased plaque macrophage lactylation, which was enhanced by exercise (Figure 3B).

HE (Figure 3C), Oil Red O (Figure 3D), and Masson staining (Figure 3E) revealed that AAV-MeCP2 K271WT treatment reinforced the utility of exercise in reducing the aortic root plaque area and increasing plaque stability, but AAV-MeCP2 K271R mice failed. AAV-MeCP2 K271WT treatment increased the serum levels of HDL cholesterol (Figure 3F). Serum levels of triglycerides (Figure 3G), total cholesterol (Figure 3H), glucose (Figure 3I), and body weight (Figure 3J) were lower in AAV-MeCP2 K271WT mice. Flow cytometry indicated that AAV-MeCP2 K271WT stimulation significantly promoted an increase in exercise-induced pro-repair of M2 macrophages, whereas AAV-MeCP2 K271R impaired it (Figure 3K). Subsequently, we analyzed the M2/M1-like genes ARG1, IL-10, INOS, and TNF-α changes in aortic root plaque. AAV-MeCP2 K271WT treatment strengthened the elevation of exercise-induced M2-like genes ARG1 and IL-10 (Figure 3L) and the decrease of M1-like genes INOS and TNF-α (Figure 3M). However, AAV-MeCP2 K271R showed the opposite results. As expected, immunofluorescence revealed that AAV-MeCP2 K271WT treatment promoted the exercise-induced infiltration of pro-repair M2 macrophages, whereas AAV-MeCP2 K271R impaired this phenomenon (Figure 3N). These results suggest that lactylation of MeCP2 K271 alters the polarization of M2 macrophages in plaques to promote atherosclerosis regression.

Therefore, to examine whether MeCP2 K271 lactylation promotes M2 macrophage polarization in vitro, we transfected BMDMs with plasmids containing FLAG-tagged MeCP2 K271WT, MeCP2 K271R, and MeCP2 K271Q and treated them with or without lactate for 24 h before harvest. Compared with that of MeCP2 K271WT mutant, MeCP2 K271R and MeCP2 K271Q mutants showed a dramatic crippling of lactate-promoting M2-like genes ARG1 and IL-10 (Figure S5A) and an intensification in lactate-inhibiting M1-like genes INOS and TNF-α (Figure S5B). Western blot suggested the similar protein expression trends of M1 macrophage markers INOS and TNF-α and M2 macrophage markers ARG1 and IL-10 in BMDMs (Figure S5C-S5E). Moreover, flow cytometry revealed that the MeCP2 K271WT mutant significantly increased the proportion of pro-repair M2 macrophages (Figure S5G) and reduced the proportion of the proinflammatory M1 macrophages (Figure S5F) in BMDMs, which was reinforced by lactate stimulation. Consistently, immunofluorescence showed that MeCP2 K271 WT mutants caused an increase in the infiltration of pro-repair M2 macrophages (Figure S5I). This resulted in a decrease in the proinflammatory M1 macrophage population (Figure S5H), which could not be prevented by lactate treatment. Collectively, these results demonstrated that exercise-induced polarization of pro-repair M2 macrophages in plaques is dominated by MeCP2 K271 lactylation.

### MeCP2 K271 lactylation interacted with H3K36me3 to attenuate atherosclerosis progression

**Figure 4:**
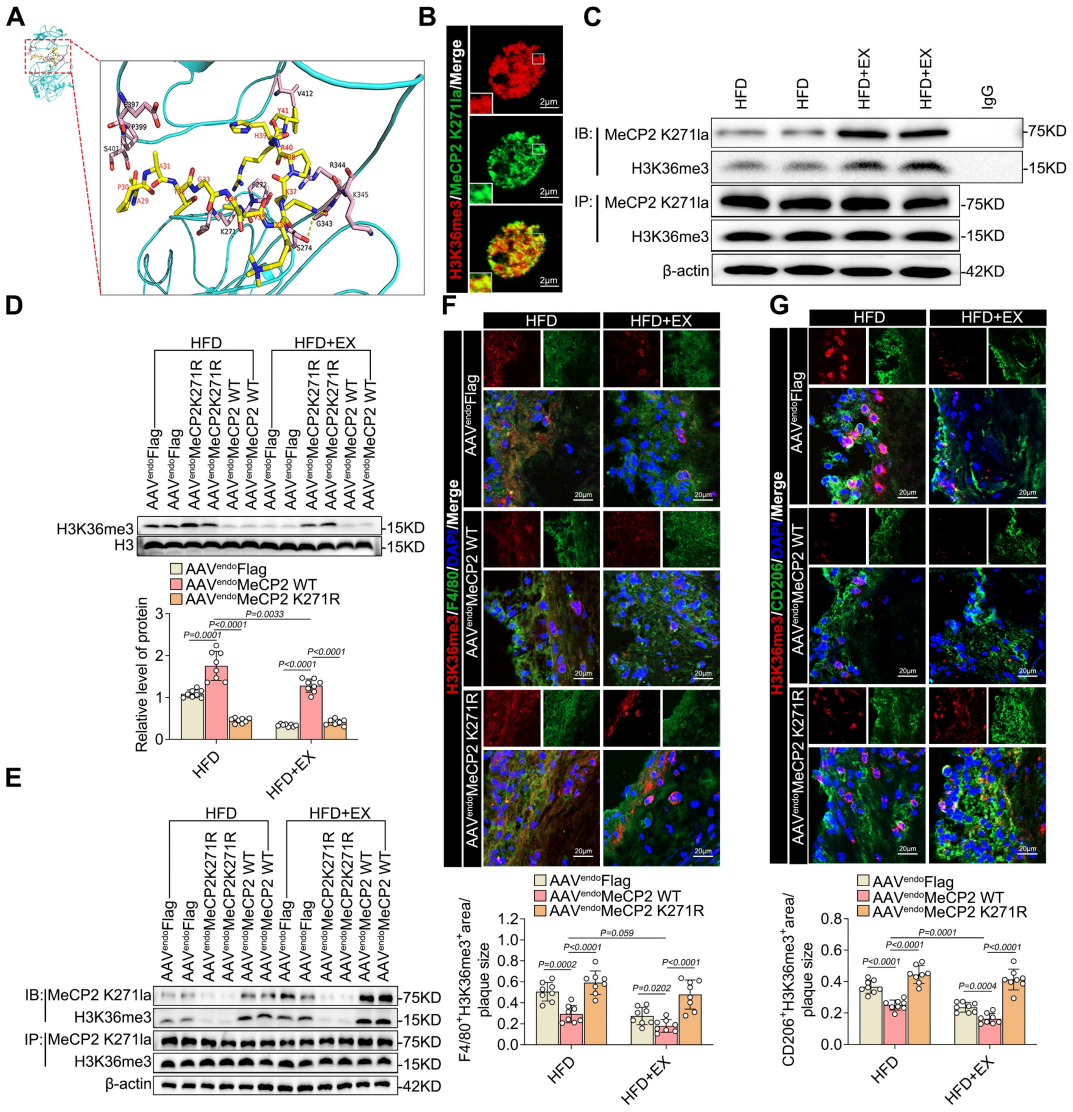
** Overexpression of MeCP2 K271 lactylation activated pro-reparative M2 macrophages by reducing the level of H3K36me3 in ApoE^-/-^ mice. A.** Overall structure of MeCP2 K271 lactylation (aquamarine) bound to H3K36me3 peptide (yellow) (left), MeCP2 K271 lactylation and peptide are shown in ribbon and rod representation, respectively (right). **B.** Representative images of confocal immunofluorescence microscopy show that MeCP2 K271 lactylation (green) colocalizes with H3K36me3 (red) in BMDMs. Scale 2 µm. **C.** Co-IP experiment for the interaction between endogenous MeCP2 K271 lactylation and H3K36me3 in isolated aortic root plaque macrophages of ApoE^-/-^ mice. **D.** Western blot analysis of protein expression level of H3K36me3 in isolated aortic root plaque macrophages of AAV-treated ApoE^-/-^ mice, with quantification of proteins. 8 mice per group. **E.** Co-IP experiment for the interaction between endogenous MeCP2 K271 lactylation and H3K36me3 isolated aortic root plaque macrophages of AAV-treated ApoE^-/-^ mice. **F, G.** Representative images of immunofluorescence co-staining for H3K36me3 together with macrophage maker F4/80 **(F)** and M2 macrophage maker CD206 **(G)** in aortic root plaques of AAV-treated ApoE^-/-^ mice with quantification of H3K36me3 intensity. 10 mice per group. Scale 20 µm. All data are shown as mean ± SD. Data were analyzed by two-tailed Student t test (A, B, C) and one-way ANOVA with Fisher's LSD post hoc test (G, I, J).

Subsequently, we investigated the effect of MeCP2 K271 lactylation on H3K36me3 levels. To gain insight into the molecular basis of H3K36me3 recognition mediated by the lactylation of MeCP2 K271, we established the structure of the H3K36me3-containing histone peptide. Structural simulation was performed to explain the strengthened interaction between H3K36me3 and MeCP2 K271 lactylation. The MeCP2 K271 lactylation model acted as a molecular pair acceptor, whereas the H3K36me3 model functioned as a molecular docking ligand. AutoDock tools were used to perform molecular docking. It was found that the lysine at K37 of H3K36me3 could form two hydrogen bonds with MeCP2 proteins S274 and G343 (Figure 4A). Accordingly, immunofluorescence staining revealed that MeCP2 K271 lactylation and H3K36me3 were colocalized in the nuclei of BMDMs (Figure 4B). We further investigated whether H3K36me3 interacts with MeCP2 K271 lactylation in HFD+EX ApoE^-/-^ mice. First, Co-IP was performed, in which MeCP2 K271 lactylation was immunoprecipitated from isolated aortic root plaque macrophages, and immunoblot analysis for H3K36me3 was conducted (Figure 3C).

These findings showed that lactylated MeCP2 K271 interacted with H3K36me3 in HFD+EX ApoE^-/-^ mice. Similar results were obtained when proteins were immunoprecipitated with an anti-H3K36me3 antibody and immunoblotted with an anti-MeCP2 K271la antibody in the HFD+EX group (Figure 3C). Subsequently, we performed further validation in ApoE^-/-^ mice with AAV-MeCP2 K271WT and AAV-MeCP2 K271R treatments. AAV-MeCP2 K271WT treatment notably downregulated the expression of H3K36me3 (Figure 3G). Immunoprecipitation with anti-H3K36me3 and anti-MeCP2 K271la antibodies revealed that AAV-MeCP2 K271R treatment disrupted the interaction between MeCP2 K271 lactylation and H3K36me3, which was not rescued by exercise (Figure 3H). Immunofluorescence also demonstrated that AAV-MeCP2 K271R treatment disrupted the exercise-induced decrease in H3K36me3 expression in F4/80 marked macrophages (Figure 3I) and CD206 marked pro-repair M2 macrophages (Figure 3J).

Subsequently, we investigated whether MeCP2 K271 lactylation and H3K36me3 have the same interactions in BMDMs. Immunoprecipitation with anti-H3K36me3 and anti-MeCP2 K271la antibodies revealed that lactate stimulation enhanced the interaction between lactylated MeCP2 K271 and H3K36me3 (Figure S6A). We then transfected BMDMs with plasmids containing FLAG-tagged MeCP2 K271 WT, MeCP2 K271R, and MeCP2 K271Q and treated them with or without lactate for 24 h. Transfection with MeCP2 K271WT resulted in an almost complete loss of H3K36me3 activity in BMDMs treated with lactate, whereas H3K36me1 and H3K36me2 were not affected (Figure S6B). The immunoprecipitation assay suggested a decrease in the interaction between lactylated MeCP2 K271 and H3K36me3; however, there were no significant changes in the interaction between lactylated MeCP2 K271, H3K9me3, and H3K27me3 (Figure S6C). To further explore the mechanism of MeCP2K271 lactylation-mediated H3K36me3, we constructed a SETD2 knockdown model in BMDMs. The loss of SETD2 led to a decrease in H3K36me3, an effect that was largely rescued by MeCP2 K271R and MeCP2 K271Q mutants. By contrast, upon treatment with MeCP2 K271 WT, knockdown of SETD2 reduced the expression of H3K36me3 (Figure S6D and S6E). We examined changes in the expression of M1/M2 macrophage marker proteins. We found that the MeCP2 K271WT mutant rescued the decrease of M2-like proteins PPAR-γ, Ym1, ARG1, and IL-10 and increase in the M1-like proteins TNF-α, COX10, IL-6, and INOS. However, the MeCP2 K271Q and MeCP2 K271R mutants failed (Figure S6D and S6E). Similarly, SETD2-knockdown BMDMs exhibited the same trends in M1/M2-like gene potential. Overexpression of MeCP2 K271Q and MeCP2 K271R disrupted these effects (Figure S6F and S6G). Flow cytometric analysis of the effect of MeCP2 K271 lactylation-mediated H3K36me3 on macrophage polarization revealed the same trend. Here, siSETD2 resulted in the upregulation of the proportion of M2 macrophages and downregulation of the percentage of M1 macrophages, and the MeCP2 K271WT mutant enhanced this phenomenon (Figure S7H and S7I). Our observations demonstrate that the activity of basic histone modifications is modulated by the crosstalk between MeCP2 lactylation and H3K36me3. The interaction between the lactylation of MeCP2 K271 and H3K36me3 favors pro-repair of M2 macrophages. Consequently, we identified the lactylation of MeCP2 K271 for the direct recognition of H3K36me3 and as a demethylation substrate of H3K36me3. In summary, exercise promotes the interaction between MeCP2 K271 lactylation and H3K36me3, thus creating an environment that facilitates H3K36me3 demethylation.

### MeCP2 K271 lactylation colocalized with H3K36me3 in chromatin accessibility remodeling that control pro-repair M2-like genes expression

**Figure 5:**
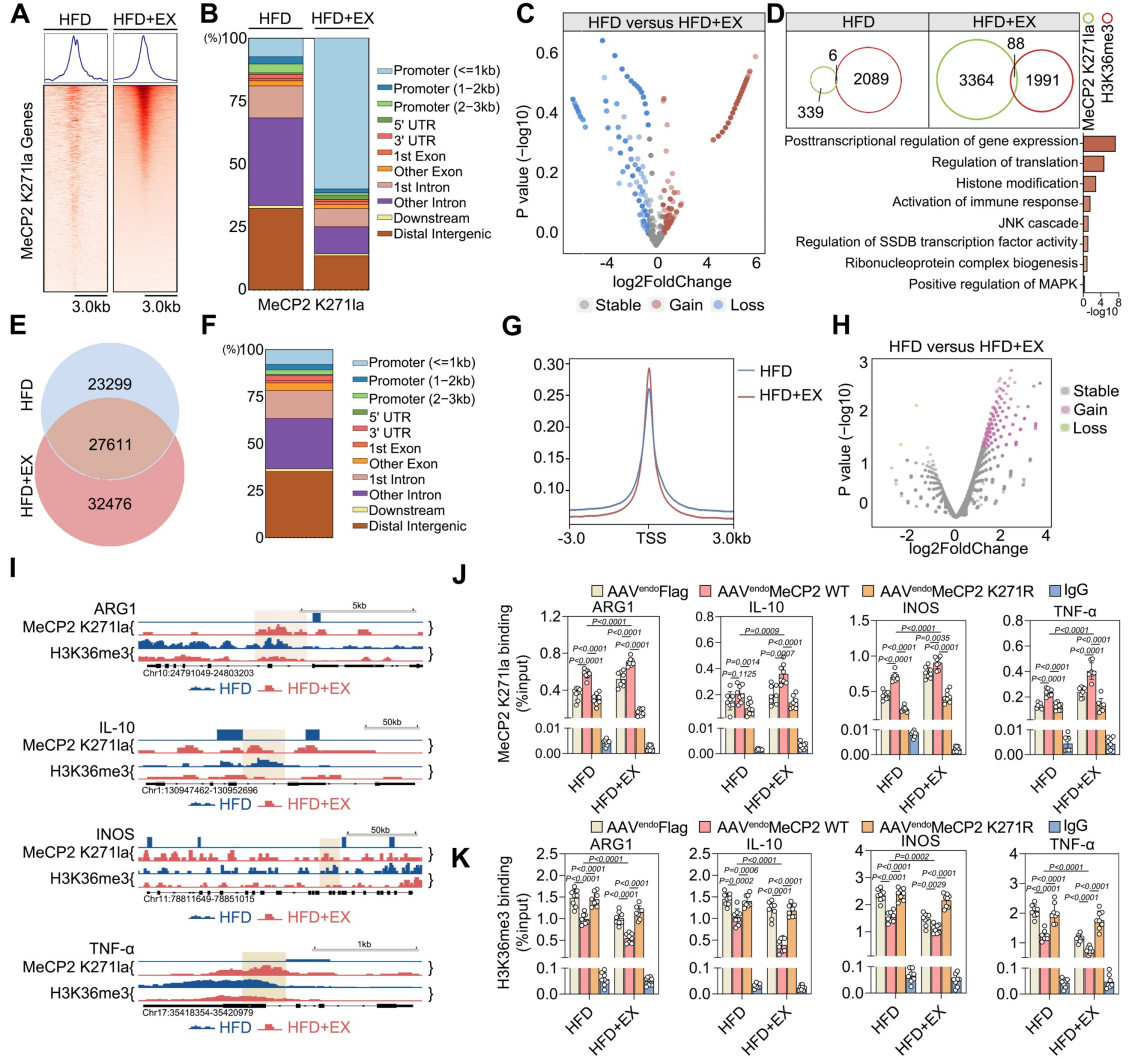
** Exercise promoted MeCP2 K271 lactylation and H3K36me3 coregulation of pro-reparative macrophage-associated genes. A.** Heatmap and averaged CUT &Tag signals of MeCP2 K271 lactylation over ±3 kb from TSS revealed the more bound transcripts from ApoE^-/-^ mice. **B.** Genome-wide distribution of MeCP2 K271 lactylation binding peaks in ApoE^-/-^ mice. **C.** Volcano plot of differential binding density of MeCP2 K271 lactylation in ApoE^-/-^ mice (loss, blue) (gain, brown) compared between the exercise and sedentary ApoE^-/-^ mice. (log2 fold change >0.58 and adj. <0.05). **D.** The Venn diagram shows the number of co-binding genes between MeCP2 K271 lactylation (green) and H3K36me3 (red) in ApoE^-/-^ mice of exercise group (top). Histogram shows the categories of co-binding between MeCP2 K271 lactylation and H3K36me3 selected from GO analysis (bottom). **E.** Venn diagram showing the number of unique and shared accessible chromatin regions in ApoE^-/-^ mice. **F.** Distribution of chromatin regions or peaks in genomic regions of ATAC-seq showing comparison between exercise and sedentary ApoE^-/-^ mice. **G.** Histogram of average chromatin accessibility of exercise and sedentary ApoE^-/-^ mice at TSS±3 kb. **H.** Volcano plot analysis of ATAC-seq density comparing exercise and sedentary ApoE^-/-^ mice. Threshold for DEGs was adj. < 0.05, log2 fold change>0.5. **I.** Integrative Genome Browser Track View for ARG1, IL-10, INOS, TNF-α genes with MeCP2 K271 lacylation/H3K36me3 CUT &Tag reads from exercise and sedentary ApoE^-/-^ mice. Regulatory regions of each gene are highlighted in blue. **J.** ChIP-qPCR of MeCP2 K271la and IgG followed by qPCR analysis at the promoters of ARG1, IL-10, INOS and TNF-α in aorta of exercise and sedentary ApoE^-/-^ mice injected with recombinant adeno-associated virus. 8 mice per group. **K.** ChIP-qPCR of H3K36me3 and IgG followed by qPCR analysis at the promoters of ARG1, IL-10, INOS and TNF-α in aorta of exercise and sedentary ApoE^-/-^ mice injected with recombinant adeno-associated virus. 8 mice per group. All data are shown as mean ± SD. Data were analyzed by one-way ANOVA with Fisher's LSD post hoc test (L, M).

To better understand the mechanism by which exercise-mediated interaction between MeCP2 K271 lactylation and H3K36me3 regulates pro-repair M2 macrophage polarization in ApoE^-/-^ mice, we performed genome-wide CUT &Tag analyses to identify candidate downstream genes in ApoE^-/-^ mice. The results showed a significant enrichment of MeCP2 K271 lactylation peaks in HFD+EX ApoE^-/-^ mice compared with those in the HFD group (Figure 5A). We also found slight enrichment of H3K36me3 peaks in the gene body (Figure S7A). A comparison revealed 3,452 MeCP2 K271 lactylation binding peaks, with 59.79% of the peaks within the promoter sequences (1 Kb) in the HFD+EX samples. In sedentary samples, there were only 345 differential MeCP2 K271 lactylation-binding peaks, of which 7.25% were within promoter sequences (Figure 5B). In contrast to the prevalence of MeCP2 K271 lactylation-binding regions in the promoter, H3K36me3 was more frequently associated with introns of transcribed genes. In addition, the enrichment of H3K36me3 binding regions in the promoter slightly increased in the HFD+EX samples (Figure S7B). We observed differential enrichment of MeCP2 K271 lactylation and H3K36me3 between the HFD+EX and HFD groups. The HFD+EX ApoE^-/-^ mice showed a loss of 863 and a gain of 2,332 binding densities for MeCP2 K271 lactylation (Figure 5C) and a loss of 1,038 and a gain of 916 binding densities for H3K36me3 (Figure S7C) compared with that of the HFD ApoE^-/-^ mice. GO analysis of MeCP2 K271 lactylation gain binding peaks of genes in the exercise animals compared with that of the sedentary group revealed that “posttranscriptional regulation of gene expression,” “nuclear transport,” and “chromatin” were the three most enriched terms (Figure S7D top). Indeed, the H3K36me3 binding loss peaks in the genes of the exercise trained ApoE^-/-^ mice were associated with the GO terms “posttranscriptional regulation of gene expression,” “mRNA processing,” and “mRNA binding” in the exercise trained group compared with that in the sedentary group (Figure S7D bottom). Integration of the results of MeCP2 K271 lactylation and H3K36me3 CUT &Tag analyses showed that the overlapping peaks were upregulated from six to 88, supporting that exercise enhances lactylation of MeCP2 K271, which is involved in the regulation of H3K36me3-directed genes (Figure 5D top). GO analysis of the coregulated genes between MeCP2 K271 lactylation and H3K36me3 was highly enriched in common terms related to “posttranscriptional regulation of gene expression,” “histone modification,” and “regulation of translation,” suggesting that the coregulated genes may be a response to the immune response in the HFD+EX ApoE^-/-^ mice (Figure 4D bottom).

Given the specific epigenetic changes in MeCP2 K271 lactylation that interact with H3K36me3 caused by exercise, we investigated the influence of exercise on changes in chromatin accessibility. We performed ATAC-seq on ApoE^-/-^ mice of the exercise and sedentary groups. We found that the accessibility of chromatin in the experimental group was increased by exercise compared with that in the sedentary group. In addition, 33% of the samples were shared between ApoE^-/-^ mice from the sedentary and exercise groups (27,611 out of 83,386 peaks). We found accessible regions that were present only during exercise (32,476 peaks) and in the sedentary samples (23,299 peaks) (Figure 5E). As expected, most of the identified open chromatin regions or ATAC peaks were located in promoters, introns, and distal intergenic regions (Figure 5F). The HFD+EX group was mainly located at or near the transcription start site compared with that of the sedentary group (Figure 5G). Global analysis of the differential accessibility of gene loci showed that exercise increased the chromatin accessibility of genes mainly related to the leukocyte migration and those responding to inflammatory stimulation (Figure 5H and S7E). GSEA of regions that became more accessible in the exercise group showed enrichment of the regulatory elements of genes associated with myeloid cell differentiation and response to inflammation (Figure S7F-S7H). Integration of CUT &Tag and ATAC-seq results showed that differentially expressed genes bound directly to MeCP2 K271 lactylation and H3K36me3, suggesting that these genes may be a secondary response to crosstalk between MeCP2 K271 lactylation and H3K36me3. This process identified four-gene in ApoE^-/-^ mice: ARG1, IL-10, INOS, and TNF-α (Figure 5I). Notably, differentially expressed genes coregulated by H3K36me3 and MeCP2 K271 lactylation were previously associated with pro-repair M2 macrophage polarization. To test whether MeCP2 K271 lactylation and H3K36me3 co-localize at the promoters of transcribed genes, we performed ChIP-qPCR in the aorta of ApoE^-/-^ mice treated with AAV-FLAG, AAV-MeCP2 K271WT, and AAV-MeCP2 K271R. We observed that AAV-MeCP2 K271WT stimulation strengthened the exercise-increased MeCP2 K271 lactylation binding at ARG1, IL-10, INOS, and TNF-α promoters (Figure 5J). Moreover, AAV-MeCP2 K271WT stimulation intensified the exercise-induced decrease in H3K36me3 binding at these promoters (Figure 5K). Collectively, these data indicate that the transcriptional regulation of MeCP2 K271 lactylation involves the regulation of H3K36me3 target genes and favors the expression of M2-associated genes.

In summary, our results reveal that exercise mediates the interaction between MeCP2 K271 lactylation and H3K36me3, which participates in chromatin accessibility remodeling and M2 macrophage polarization.

### MeCP2 K271 lactylation improved pro-reparative M2 macrophage polarization through reduction of RUNX1 expression

**Figure 6:**
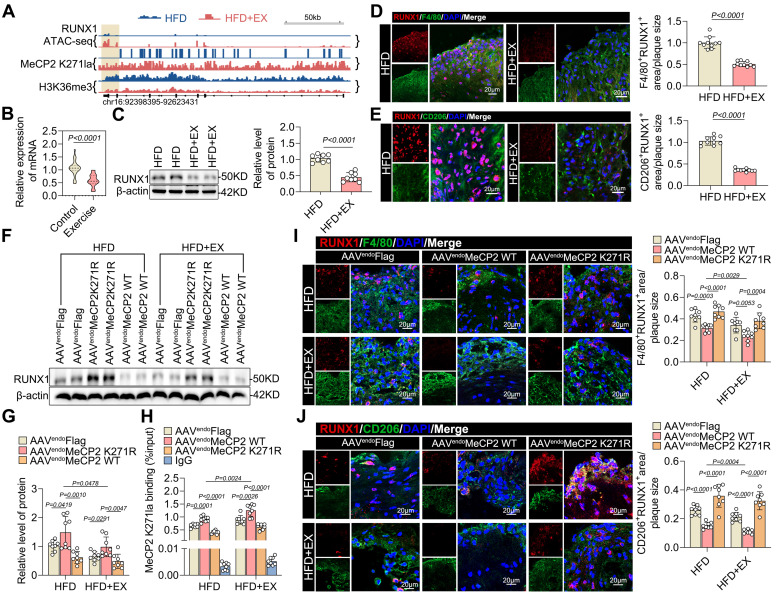
** Overexpression of MeCP2 K271 lactylation disrupted RUNX1 expression in ApoE^-/-^ mice. A.** Integrative Genome Browser Track View for RUNX1 gene with ATAC-seq, MeCP2 K271 lacylation/H3K36me3 CUT &Tag reads from exercise and sedentary ApoE^-/-^ mice. Regulatory regions of each gene are highlighted in blue. **B.** qRT-PCR analysis of RUNX1 expression in isolated PBMCs from patients with atherosclerosis treated with or without physical exercise. 30 patients per group. **C.** Western blot analysis of RUNX1 levels in isolated aortic root plaque macrophages of ApoE^-/-^ mice with quantification of protein levels. 8 mice per group. **D, E.** Representative images of immunofluorescence co-staining for RUNX1 together with macrophage maker F4/80 (D) and M2 macrophage marker CD206 (E) in aortic root plaques from ApoE^-/-^ mice, with quantification of RUNX1 intensity. 10 mice per group. Scale 20 µm. **F, G, H.** Western blot analysis of protein expression level of RUNX1 in isolated aortic root plaque macrophages of ApoE^-/-^ mice transfected with indicated AAV, with quantification of proteins. 8 mice per group. **I, J.** Representative images of immunofluorescence co-staining for RUNX1 together with macrophage F4/80 (H) and M2 macrophage maker CD206 (I) in aortic root plaques from ApoE^-/-^ mice transfected with indicated AAV, with quantification of RUNX1 intensity. 10 mice per group. Scale 20 µm. All data are shown as mean ± SD. Data were analyzed by two-tailed Student t test (B, C, D) and one-way ANOVA with Fisher's LSD post hoc test (G, H, J, K).

To determine which transcription factors might mediate exercise-alleviated atherosclerosis, we integrated the CUT &Tag and ATAC-seq data and examined the distribution of MeCP2 K271 lactylation and H3K36me3 modifications in relation to regions of open chromatin identified by ATAC-seq in exercised and sedentary ApoE^-/-^ mice. There was a significant association between RUNX1 and the interactions between MeCP2 K271 lactylation and H3K36me3, as well as the opening of chromatin accessibility (Figure 6A). Recent studies have also suggested that RUNX1 is involved in the downstream effects of hyperglycemia-induced trained immunity in macrophages 36. Therefore, we hypothesized that RUNX1 is epigenetically regulated by exercise in atherosclerosis. Subsequently, we examined the effects of exercise on atherosclerosis. Our qRT-PCR results also showed that the mRNA expression of RUNX1 was lower in PBMCs from exercise-treated patients with ASCVD than in the control sample (Figure 6B). The baseline characteristics of the study population are presented in Table S2. Western blot analysis revealed that RUNX1 levels were downregulated in isolated aortic root plaque macrophages of the HFD+EX group (Figure 6C). Co-staining of plaques for RUNX1 and the macrophage marker F4/80 showed a twofold decrease in the exercise group compared with that in the sedentary group (Figure 6D), indicating that exercise inhibited the expression of RUNX1 in macrophages. Moreover, co-staining of plaques for RUNX1 and the M2 macrophage marker CD206 showed a significant decrease in HFD+EX mice compared with that in HFD mice (Figure 6E), indicating that RUNX1 affects the polarization of pro-repair M2 macrophages. To determine whether the exercise-mediated decrease in RUNX1 expression was regulated by the MeCP2 K271 lactylation-H3K36me3 pathway, we performed further validation in ApoE^-/-^ mice treated with AAV-FlAG, AAV-MeCP2 K271WT, or AAV-MeCP2 K271R. We observed that AAV-MeCP2 K271WT stimulation enhanced exercise-induced inhibition of RUNX1, whereas AAV-MeCP2 K271R stimulation failed (Figure 6F and 6G). ChIP-qPCR analysis confirmed that AAV-MeCP2 K271WT treatment strengthened exercise-induced inhibition of MeCP2 K271 lactylation enrichment at the RUNX1 promoter, whereas AAV-MeCP2 K271R treatment remained unaffected (Figure 6H). Consistently, immunofluorescence showed that AAV-MeCP2 K271WT treatment caused an additional decrease in RUNX1 expression in the aortic plaque macrophages of ApoE^-/-^ mice, particularly in pro-repair M2 macrophages; however, AAV-MeCP2 K271R treatment did not (Figure 6I and 6J). To determine the RUNX1 consequences of MeCP2 K271 lactylation-mediated H3K36me3 expression, we used the BMDMs model. ChIP-qPCR analysis showed that MeCP2 K271 lactylation and H3K36me3 were enriched in the RUNX1 promoter after treatment with lactate (Figure S8A and S8B). Moreover, ChIP-qPCR analysis confirmed that the MeCP2 K271Q or K271R mutants reduced RUNX1 promoter enrichment in lactate-treated BMDMs (Figure S8C). Knockdown of SETD2 in BMDMs led to a decrease in the expression of RUNX1, an effect that could be largely rescued by the overexpression of MeCP2 K271R or MeCP2 K271Q (Figure S8D and S8E). Immunostaining of RUNX1 together with the M1 macrophage marker CD86 and M2 macrophage marker CD206 in BMDMs with the indicated treatments suggested that siSETD2 decreased the proportion of M1 macrophages and increased the percentage of M2 macrophages, and the MeCP2 K271WT mutant intensified these effects (Figure S8F). Overall, our results suggest that the increased expression of RUNX1 in plaques may be detrimental, and that exercise inhibits the expression of RUNX1, which contributes to the polarization of pro-repair M2 macrophages.

Collectively, these results suggest that crosstalk between MeCP2 K271 lactylation and H3K36me3 is required for the regulation of RUNX1 to induce the pro-repair of M2 macrophage polarization.

### Pharmacological inhibition of RUNX1 reduced atherosclerotic plaque development and was essential for M2 macrophage polarization

**Figure 7:**
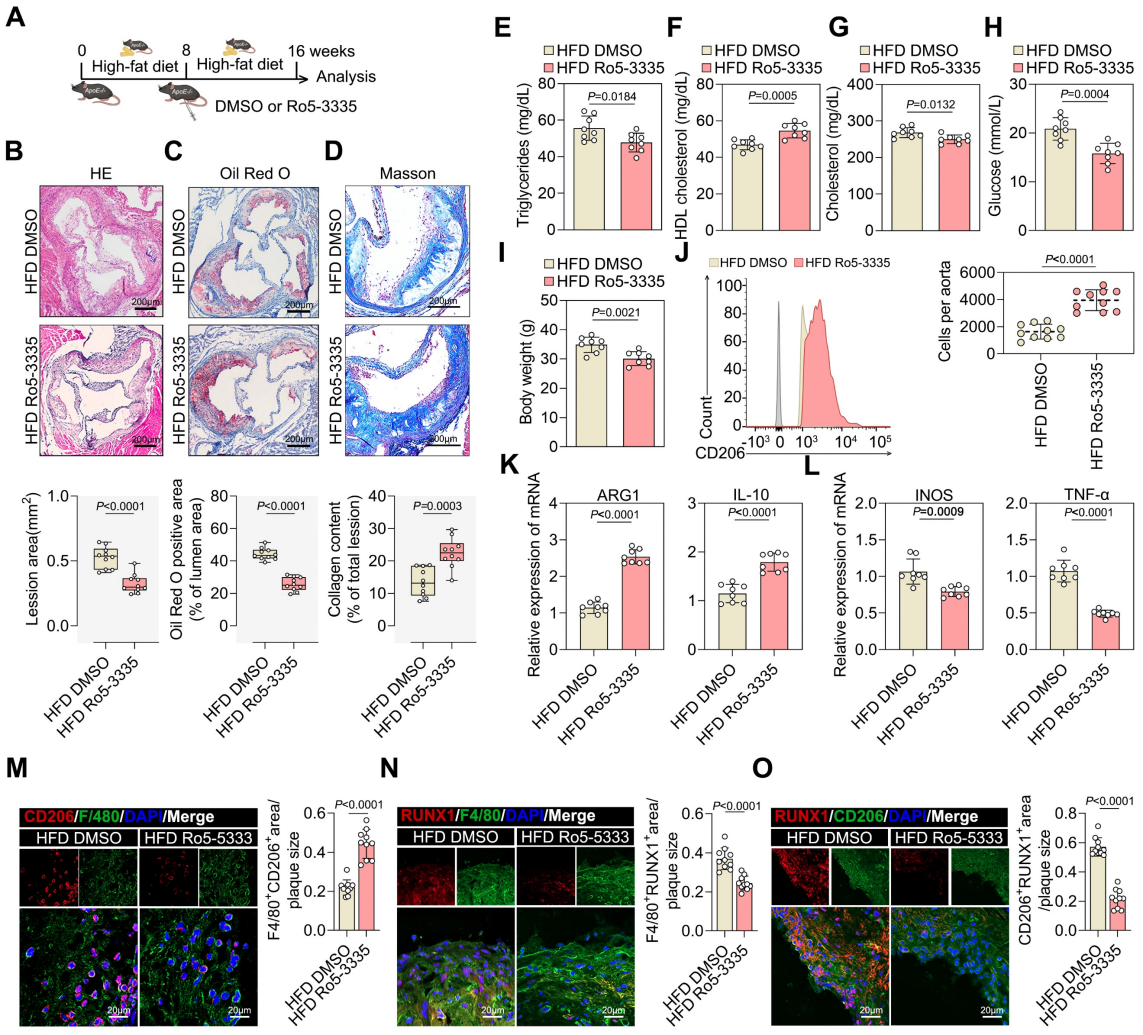
** Pharmacological inhibition of RUNX1 reduced atherosclerotic plaque development and increased pro-reparative M2 macrophage accumulation in ApoE^-/-^ mice. A.** Experimental outline. ApoE^-/-^ mice receiving HFD for 8 weeks were treated with Ro5-3335 (25 mg/kg) or DMSO starting at week 8. **B, C, D.** Representative images (top) and quantification (bottom) of atherosclerotic area in cross-sections of aortic root of ApoE^-/-^ mice treated with Ro5-3335 and DMSO by HE **(B)**, Oil Red O **(C)** and Masson staining **(D)**. Scale 200 μm; 10 mice per group. **E-I.** Measurement of atherosclerosis protective/risk factor levels: serum HDL cholesterol **(F)**, serum triglycerides **(E)**, serum cholesterol **(G)**, serum glucose **(H)** and body weight** (I)**. 8 mice per group. **J, K, L.** Representative flow cytometric plots **(J)** and quantification **(K, L)** of pro-repair M2 macrophage populations in the aotric root plaque from ApoE^-/-^ mice treated with Ro5-3335 and DMSO. 8 mice per group. **M.** Representative immunofluorescence co-staining images for macrophage maker F4/80 and M2 macrophage maker CD206 in aortic root plaques of ApoE^-/-^ mice treated with Ro5-3335 and DMSO, with quantification of CD206 intensity. 10 mice per group. **N, O.** Representative images of immunofluorescence co-staining for RUNX1 together with macrophage maker F4/80 and M2 macrophage marker CD206 in aortic root plaques from ApoE^-/-^ mice treated with Ro5-3335 and DMSO, with quantification of immunofluorescence intensity. 10 mice per group. n represents number of independent samples. All data are shown as mean ± SD. All data were analyzed two-tailed Student t test.

Accordingly, we investigated the therapeutic effects of the RUNX1 inhibitor, Ro5-3335, in established atherosclerosis mouse models. ApoE^-/-^ mice fed a FHD for eight weeks were treated with Ro5-3335 or DMSO for eight weeks (Figure 7A). Ro5-3335 attenuated the progression of established atherosclerosis, as evidenced by a reduction in lesion size compared with that in DMSO-treated ApoE^-/-^ mice (Figure 7B and 7C). The relative levels of collagen increased in Ro5-3335 treated ApoE^-/-^ mice (Figure 7D). Furthermore, Ro5-3335 stimulation also reduced the levels of the atherosclerosis risk factors serum triglycerides, serum cholesterol, serum glucose, and body weight, while significantly elevating the level of the atherosclerosis protective factor serum HDL (Figure 7E-7I). Flow cytometry analysis suggested that Ro5-3335 induced the pro-repair of M2 macrophages in isolated aortic root plaques (Figure 7J). Additionally, Ro5-3335 increased the expression of M2-like genes ARG1 and IL-10 (Figure 7K) and decreased the expression of M1-like genes INOS and TNF-α (Figure 7L) in isolated aortic root plaque macrophages. Moreover, Ro5-3335 increased the area of pro-repair M2 macrophage infiltration in plaques compared with that in DMSO-treated ApoE^-/-^ mice (Figure 7M). Co-staining of plaques with RUNX1 and the macrophage marker F4/80 showed lower RUNX1 levels in Ro5-3335-treated ApoE^-/-^ mice compared with those in DMSO-treated ApoE^-/-^ mice (Figure 7N). Co-staining for RUNX1 with the M2 macrophage marker CD206 in plaques revealed a threefold decrease in the Ro5-3335-treated group compared with that in the HFD ApoE^-/-^ mice (Figure 7O). The results showed that Ro5-3335 was sufficient to induce macrophage polarization towards a pro-repair state in the plaques.

Collectively, these results demonstrate that the pharmacological inhibition of RUNX1 promotes pro-repair M2 macrophage polarization in plaques, increases lesion stability, and improves the prognosis of ASCVD.

## Discussion

MeCP2 is known for its role in the organization of chromatin structures, formation of chromatin loops, and the recruitment of auxiliary repressors and activators 24, 25. It has been shown that MeCP2 regulates gene transcription through additional interactions with chromatin, such as posttranslational modifications in proteins. However, further studies are required to elucidate the role of MeCP2 interactions with PTMs in the regulation of gene transcription in diseases. In this study, we found that exercise leads to the lactylation of MeCP2 K271, which facilitates the interaction between MeCP2 K271 lactylation and H3K36me3, and is involved in chromatin accessibility changes that transcriptionally regulate a number of macrophage polarization-associated genes. Our results also showed that the downstream target gene RUNX1 acts as a mediator of pro-repair M2 macrophage polarization. Pharmacological inhibition of RUNX1 ameliorates progression of atherosclerotic plaques.

MeCP2 is one of the central readers of the epigenome in both normal and pathophysiological contexts 37, 38. MeCP2 mutations have been designated as mutational "hotspots" associated with neurodevelopmental disorders, such as Rett syndrome 39, autism 40, 41, and Angelman-like syndrome 42. Phosphorylation of S421 was the first posttranslational modification of MeCP2 to be described and was found to be induced by increased neuronal activity associated with calcium ion influx 28. Moreover, the phosphorylation of MeCP2 sites (S86, S274, and T308) is induced by neuronal activity, brain-derived neurotrophic factors, or agents that elevate the intracellular levels of cAMP, suggesting that MeCP2 may function as an epigenetic regulator of gene expression that integrates various signals from the environment 29. Consistent with this possibility, in our exercise model using HFD ApoE^-/-^ mice, increased lactate levels in the internal environment promoted one aspect of exercise-dependent lactylation of MeCP2 at K271. Moreover, when BMDMs were stimulated with lactate in vitro, MeCP2 was lactylated at K271. MeCP2 K271 site-mutant mice were generated by AAV transfection. These findings suggest that the lactylation of MeCP2 is K271 site-specific. Unsurprisingly, the MeCP2 K271R mutant failed to perpetuate the ability of exercise to promote pro-repair of M2 macrophage polarization to improve atherosclerosis. Similarly, in vitro experiments demonstrated that MeCP2 K271 lactylation promoted the polarization of BMDM towards pro-repair M2 macrophages, whereas the MeCP2 K271Q and MeCP2 K271R mutants failed. Collectively, our study confirmed that exercise-mediated MeCP2 K271 lactylation promotes macrophage polarization towards the pro-repair M2 phenotype, which, in turn, exerts anti-atherosclerotic effects.

MeCP2 has histone deacetylation and methylation-binding partners that regulate its ability to bind and organize chromatin 43. It is also considered essential for higher-order/long-range chromatin remodeling and silencing, with roles in heterochromatin formation and chromatin organization 44. MeCP2 can recruit HDAC3 to mediate the remodeling of inactive chromatin or directly induce chromatin compaction to repress gene expression 45. Interestingly, our results show that HDAC3 is an efficient delactylase for MeCP2 K271 lactylation, supporting the novel role of HDAC3 in the dynamic regulation of MeCP2. Evidence suggests that H3K36me3 plays a critical role in partitioning chromatin into specific domains and demarcating the body regions of actively transcribed genes, thus providing signals to modulate transcriptional fidelity 46. The regulation of H3K36 methylation marks is biologically critical and controlled by different protein classes. Significant efforts have been made to identify the enzymes that mediate the demethylation of H3K36. Histone demethylation has drawn attention because of its potential role in regulating immune responses 47-49. Histone demethylation of JMJD3 enhances the polarization of pro-repair M2 macrophages following IL-4 treatment and directly regulates the Arg1 promoter 50. Exercise decreased H3K36me3 expression in HFD ApoE^-/-^ mice. Meanwhile, we identified exercise-induced MeCP2 lactylation at the K271 site as a specific and direct "recognizer" of H3K36me3. MeCP2 K271 lactylation recognition of H3K36me3 methylation is a key event in MeCP2 lactylation for histone demethylation. The evidence presented here, following AAV-transfection of MeCP2 K271WT, indicates a greater decrease in H3K36me3 expression, enabling the polarization of macrophages to pro-repair macrophages. In conclusion, our study demonstrated that lactylated MeCP2 at K271 specifically binds to histones and coregulates gene transcription by affecting methylation.

Targeted lactylation of MeCP2 at K271 interacting with H3K36me3-associated signaling represents an attractive strategy for the treatment of ASCVD. It is evident that the lactylation of MeCP2 at K271 plays a role in coordinating the crosstalk between non-histone lactylation and H3K36me3, which is conserved in the effect of exercise on ASCVD. This crosstalk leads to a specific H3K36 modification, and this "combinatorial histone language" extends its scope of meaning to the chromatin landscape. In particular, the lactylation of MeCP2 at K271-mediated recognition of H3K36me3 is critical for the optimal silencing of gene expression programs closely associated with the regulation of macrophage polarization. We found that exercise positively regulated the transcription of a subset of macrophage polarization genes and regulated MeCP2 K271 lactylation, which interacts with H3K36me3 on the promoters of M2-associated genes. Exercise-induced specific chromatin accessibility was upregulated, and MeCP2 K271 lactylation was significantly enriched with H3K36me3 changes at genomic transcription start sites. To investigate the nature and extent of potential changes in these regulatory layers and their impact on exercise-induced specific gene expression, we subjected ApoE^-/-^ mice to integrated analysis using ATAC-seq and CUT &Tag analysis. Our data suggest that increased chromatin accessibility is a novel defining feature of exercise-induced anti-atherosclerosis that contributes to the regulation of gene expression in this disease. Using integrated genomic profiling, we demonstrated that exercise facilitated the binding of MeCP2 K271 lactylation to H3K36me3-marked loci, thereby enhancing gene silencing. We found that exercise-induced specific chromatin accessibility was upregulated, and MeCP2 K271 lactylation was significantly enriched with H3K36me3 changes at the genomic transcription start sites. This enrichment was observed in RUNX1 regions, which became more accessible in the exercise group ApoE^-/-^ mice, indicating substantial chromatin reconfiguration.

RUNX1, a member of the core-binding family of transcription factors, is involved in the cell cycle, proliferation, ribosome genesis, apoptosis, and immune regulation during normal development and disease 51. Transcriptomes of macrophages in atherosclerotic plaques and peripheral leukocytes from patients with type 2 diabetes were enriched in RUNX1 targets, and targeting RUNX1 provided further evidence for the involvement of RUNX1 in the downstream effects of hyperglycemia-induced trained immunity in macrophages 36. A study on inducible cardiomyocyte-specific RUNX1 deficiency in mice demonstrated that RUNX1 deficiency protected against adverse cardiac remodeling after myocardial infarction 36, 52. Our study shows that RUNX1 is essential for exercise-mediated effects on the polarization of pro-repair M2 macrophages. Our results using the RUNX1 inhibitor Ro5-3335 demonstrated that blocking RUNX1 attenuated the progression of atherosclerotic plaques and promoted macrophage polarization towards pro-repair M2 macrophages, which led to an increase in plaque stability. Therefore, targeting RUNX1 may be a novel treatment option for ASCVD.

We demonstrated that exercise is effective in improving the progression of atherosclerotic plaque. However, we focused on lactylation changes under exercise and non-exercise conditions in high-fat mice and did not investigate whether exercise in non-high-fat mice would cause lactylation changes. In the experimental animal model, we used adenoviral interference, which may be incomplete, so the specificity of the altered locus could be better demonstrated by creating synthetic mutant animals using CRISPR/Cas9 technology. In future studies, researchers can focus on the regulatory effects of MeCP2 lactylation on non-histone proteins and find more downstream regulation of MeCP2 lactylation modification. And several studies have reported that MeCP2 bind to histone methylation at the transcription start site to co-regulate gene transcription 53. We also question whether lactate-mediated MeCP2 K271 lactylation affects the interaction between MeCP2 with other histone methylation. In addition, MeCP2 is a key molecule in the regulation of nervous system development, and future studies should continue to explore whether MeCP2 lactylation can provide new insights into the treatment of Rett syndrome.

For future exercise-targeted therapies, it is important to identify and characterize the posttranslational modifications of non-histones in ASCVD. Overall, our study uncovered the specific mechanism of the posttranslational modification of non-histone MeCP2 involved in regulating the polarization of macrophages to pro-repair M2 macrophages. This may help explain how exercise affects macrophages, promoting plaque stability, and improving the prognosis of atherosclerosis. Crosstalk between the lactylation of MeCP2 K271 and H3K36me3 is involved in chromatin regulation and allows for increased regulation of transcription genes. The identification of specific epigenetic modifications and targets of RUNX1 suggests potential new therapeutic targets for exercise.

## Supplementary Material

Supplementary figures and tables.

## Abbreviations

ASCVD: Atherosclerotic cardiovascular disease
HFD: High fat diet
HFD+EX: High fat diet+exercise
Kla: Lysine lactylation
MeCP2: Methyl CpG binding protein 2
RUNX1: Runt-related transcription factor 1
SETD2: Histone methyltransferase Set2
WT: Wild type
