# Supplementary material for: Methyl-CpG-binding 2 K271 lactylation-mediated M2 macrophage polarization inhibits atherosclerosis
Source: Theranostics. 2024 Jul 8;14(11):4256–77. doi: 10.7150/thno.94738 (PMC11303070; doi:10.7150/thno.94738)
Supplement: Supplementary file 1 — Supplementary figures and tables. [file thnov14p4256s1.pdf]

Supplemental Data for

Methyl-CpG-binding 2 K271 lactylation-mediated M2 macrophage polarization inhibits atherosclerosis

Running Title: Liangqi Chen, et al.; Methyl-CpG-binding 2 K271 lactylation inhibits atherosclerosis

Liangqi Chen[1,2*], Meiju Zhang[1,2*], Xueyan Yang[1,2*], Yanan Wang[1,2], Tuo Huang[1,2], Xin Li[1,2], Yunting Ban[1,2], Qifeng Li[1,2], Qingyuan Yang[1,2], Yongxiang Zhang[1,2], Yang Zheng[1,2], Di Wang[1,2], Xiaoqi Wang[1,2], Xiujie Shi[4], Maomao Zhang[1,2], Yong Sun[1,2], Jian Wu[1,2,3**]

[1]Department of Cardiology, The Second Affiliated Hospital of Harbin Medical University, Harbin, China;

[2]The Key Laboratory of Myocardial Ischemia, Chinese Ministry of Education, Harbin, China;

[3]Cardiac Rehabilitation Center, Department of Cardiology, The Second Affiliated Hospital of Harbin Medical University, Harbin, China;

[4]The Clinical Skills Center, The Second Affiliated Hospital of Harbin Medical University, Harbin, China;

[*]These authors contributed equally to this work

**** Address for Correspondence:**

Department of Cardiology

The Second Affiliated Hospital of Harbin Medical University

Xuefu Road, Harbin, China

Email:

Jian Wu: wujian780805@163.com

Tel: 0451-86605465

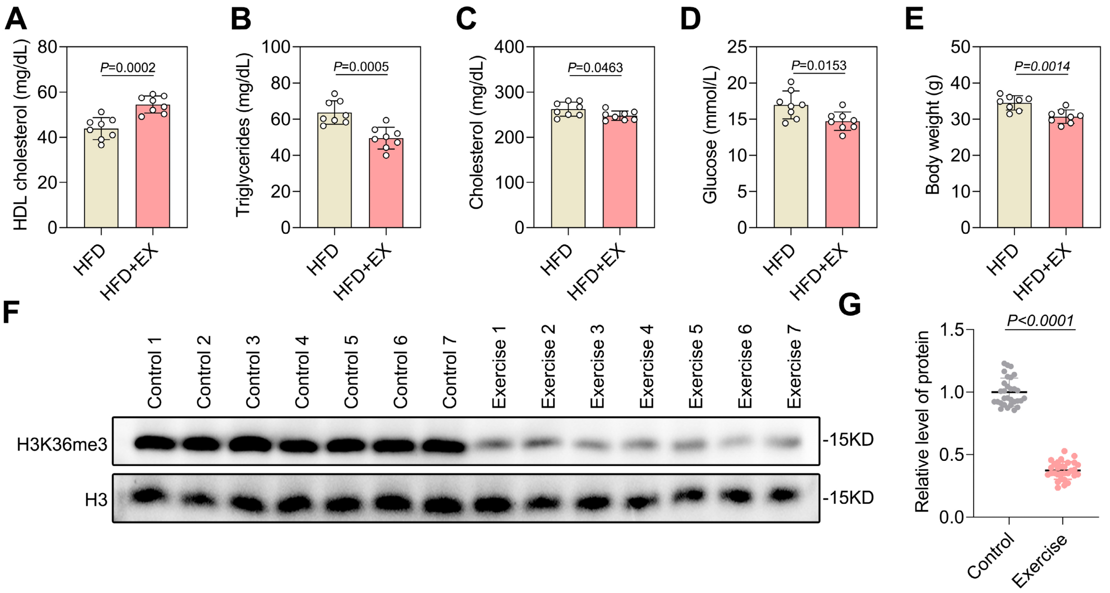

Figure S1. Exercise lowered risk factors for atherosclerosis and created an demethylated environment in ApoE$^{-/-}$ mice.

A-E. Measurement of atherosclerosis protective/risk factor levels: serum HDL cholesterol **(A)**, serum triglycerides **(B)**, serum cholesterol **(C)**, serum glucose **(D)** and body weight **(E)**. 8 mice per group. **F.** Western blot analysis of H3K36me3 expression in isolated PBMCs from patients with atherosclerosis treated with or without exercise training. 30 patients per group. n represents number of independent samples. All data are shown as mean±SD. Data were analyzed by two-tailed Student t test.

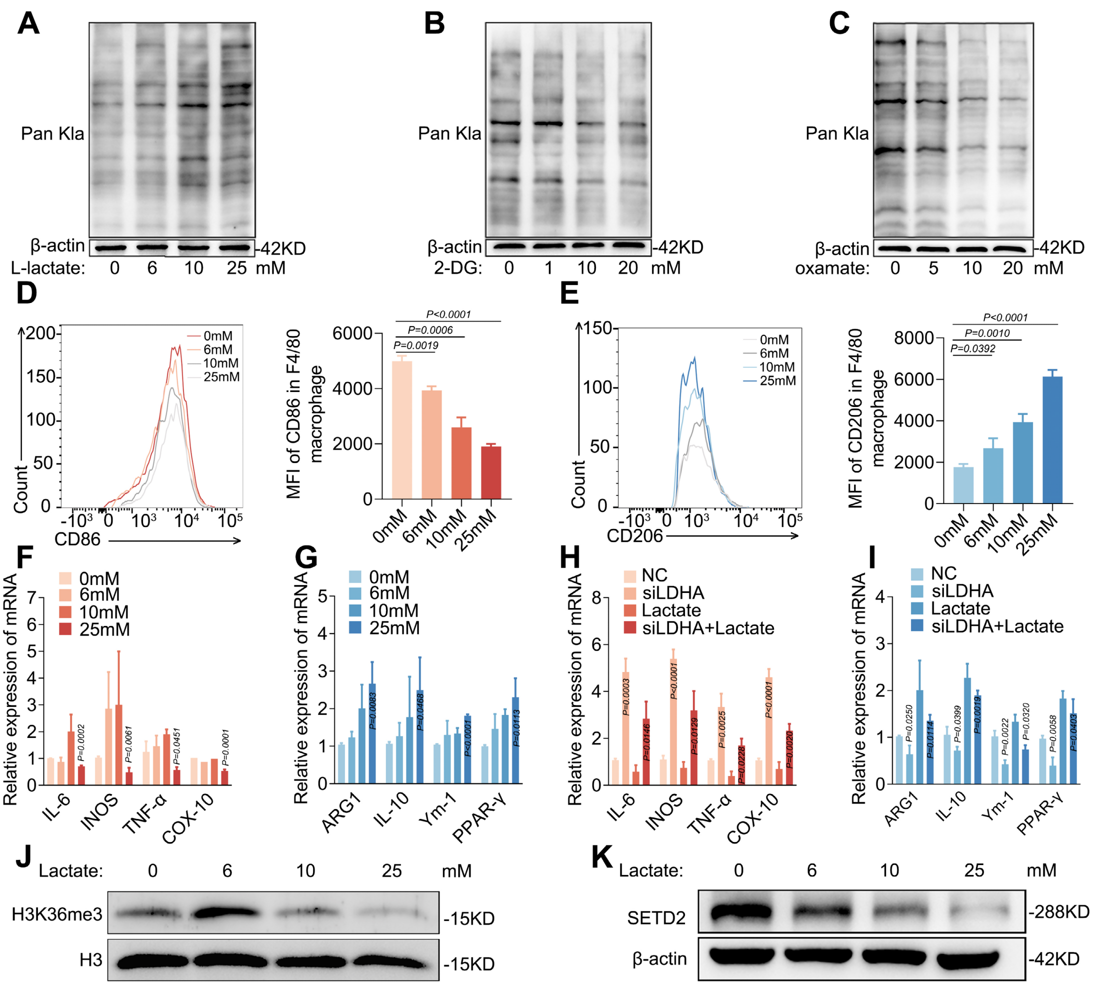

Figure S2. Lactate induces the phenotype of M2 macrophage in BMDMs through Pan Kla.

A. Western blot analysis of Pan Kla level in BMDMs cultured in different lactate concentrations. **B.** Western blot analysis of Pan Kla in BMDMs cultured in different concentrations of 2-DG. **C.** Western blot analysis of Pan Kla in BMDMs cultured in different concentrations of oxamate. **D.** Representative flow cytometric plots of M1 macrophage marker CD86 and mean fluorescence intensity of CD86 macrophages in BMDMs stimulated with different concentrations of lactate. **E.** Representative flow cytometric plots of M2 macrophage marker CD206 and mean fluorescence intensity of CD206 macrophages in BMDMs stimulated by different lactate concentrations. **F, G.** qRT-PCR analysis of M1-like genes **(F)** and M2-like genes **(G)** in BMDMs stimulated by different lactate concentrations. **H, I.** qRT-PCR analysis of M1-like genes **(H)** and M2-like genes **(I)** in BMDMs treated with control (siNC) or LDHA siRNA and lactate for 24 h. n represents number of independent samples. **J.** Western

blot analysis shows that lactate decreases expression of H3K36me3 in BMDMs. **K.** Western blot analysis of SETD2 protein levels in BMDMs treated with lactate. All data are shown as mean±SD. Data were analyzed by one-way analysis ANOVA with Fisher's LSD post hoc test.

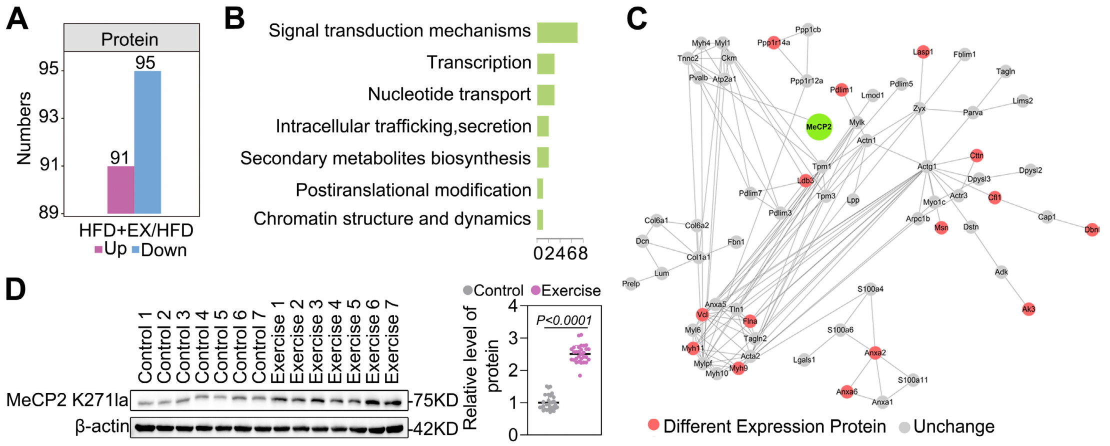

Figure S3. Lactylation of MeCP2 K271 was upregulated during exercise.

A. Numbers of protein exhibiting remarkable changes in levels induced by exercise. **B.** Gene Ontology terms representing function of proteins exhibiting remarkable Kla changes or remarkable changes in levels. **C.** PPI analysis of nuclear chromosome-associated Kla proteins based on the database STRING. **D.** Western blot analysis of MeCP2 K271 lactylation expression in isolated PBMCs from patients with atherosclerosis treated with or without exercise training. 30 patients per group. All data are shown as mean±SD. Data were analyzed by two-tailed Student t test.

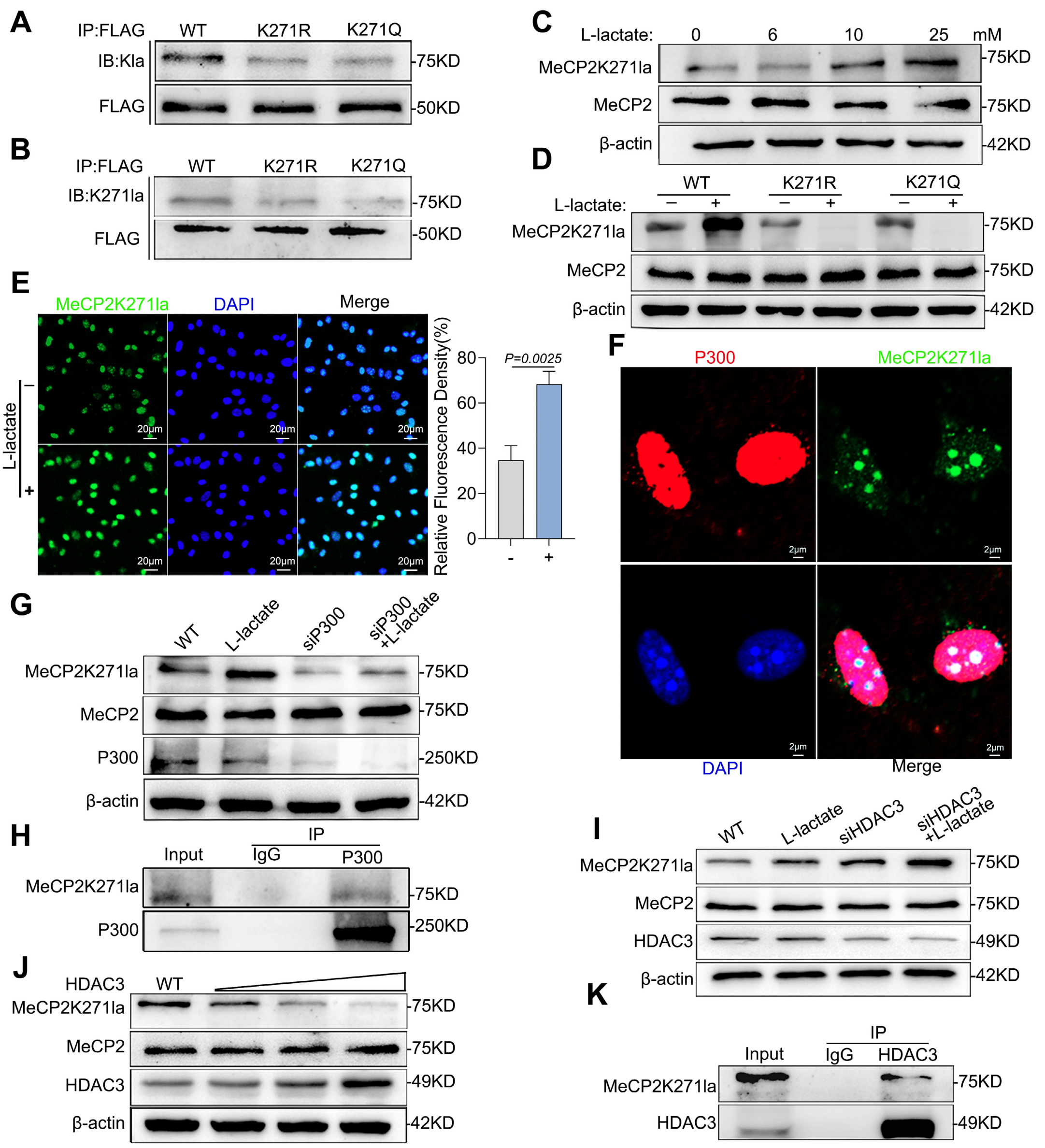

Figure S4. P300 and HDAC3 are involved in the regulation of lactate-induced lactylation of MeCP2 K271 in BMDMs.

A, B. Western blot analysis of Pan Kla **(A)** and MeCP2 K271 lactylation **(B)** levels in BMDMs transfected with FLAG-tagged MeCP2 K271WT, MeCP2 K271Q and MeCP2 K271R. **C.** Western blot analysis of MeCP2 K271 lactylation level in BMDMs cultured with lactate for gradient concentration. **D.** Western blot analysis of MeCP2 K271 lactylation level in BMDMs treated with FLAG-tagged MeCP2 K271WT, MeCP2 K271Q and MeCP2 K271R plasmids and lactate for 24 h. **E.** Immunofluorescence analysis of MeCP2 K271 lactylation (green) in BMDMs treated with lactate. DAPI (blue), cell nuclei. Scale 20 μm. **F.** Representative images of

confocal immunofluorescence microscopy show that MeCP2 K271 lactylation (green) colocalizes with P300 (red) in BMDMs. DAPI (blue), cell nuclei. Scale 2 μm. **G.** Western blot analysis of MeCP2 K271 lactylation and P300 levels in BMDMs treated with control (siNC) or P300 siRNA and lactate for 24 h. **H.** Immunoprecipitation with anti-IgG and anti-P300 antibodies and immunoblots analysis with anti-MeCP2K271la antibody. **I.** Western blot analysis of MeCP2 K271 lactylation and HDAC3 levels in BMDMs treated with control (siNC) or HDAC3 siRNA and lactate for 24 h. **J.** Western blot analysis of MeCP2 K271 lactylation and HDAC3 levels in BMDMs treated with lactate for gradient concentration. **K.** Immunoprecipitation with anti-IgG and anti-HDAC3 antibodies and immunoblots analysis with anti-MeCP2K271la antibody. All data are shown as mean±SD. Data were analyzed by two-tailed Student t test.

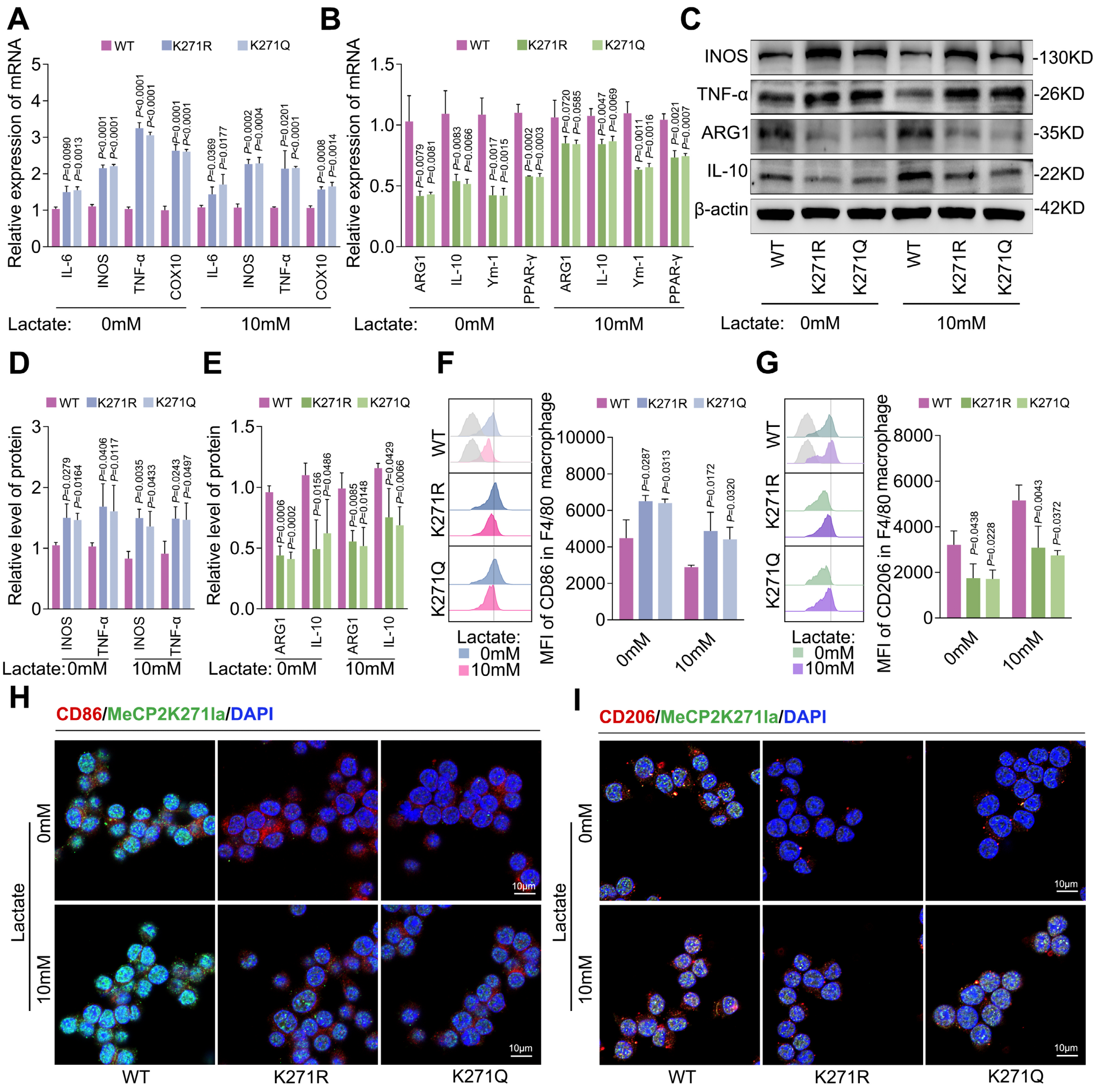

Figure S5. MeCP2 K271 lactylation is associated with accumulation of pro-repair M2 macrophages in BMDMs.

A. qRT-PCR analysis of M1-like genes in BMDMs transfected with the indicated plasmids and treated with or without stimulation of lactate. **B.** qRT-PCR analysis of M2-like genes in BMDMs transfected with the indicated plasmids and treated with or without stimulation of lactate. **C-E.** Western blot images showing the protein levels of ARG1, IL-10, INOS and TNF-α in BMDMs transfected with indicated plasmids and incubated with or without lactate **(C)** and its quantification **(D, E)**. **F.** Representative flow cytometric plots of M1 macrophage marker CD86 and mean fluorescence intensity of CD86 macrophages in BMDMs were transfected with the indicated plasmids and were treated with or without stimulation of lactate. **G.** Representative flow cytometric plots of M2 macrophage marker CD206 and mean fluorescence

intensity of CD206 macrophages in cells were transfected with the indicated plasmids and were treated with or without stimulation of lactate. **H.** Immunofluorescence analysis with with anti-MeCP2K271la and anti-CD86 antibodies in BMDMs transfected wit indicated plasmids and treated with or without stimulation of lactate for 24 h. Scale 10μm. **I.** Immunofluorescence analysis with with anti-MeCP2K271la and anti-CD206 antibodies in BMDMs transfected wit indicated plasmids and treated with or without stimulation of lactate for 24 h. Scale 10μm. Results are representative of three or more independent experiments. All data are shown as mean±SD. Data were analyzed by one-way ANOVA with Fisher's LSD post hoc test.

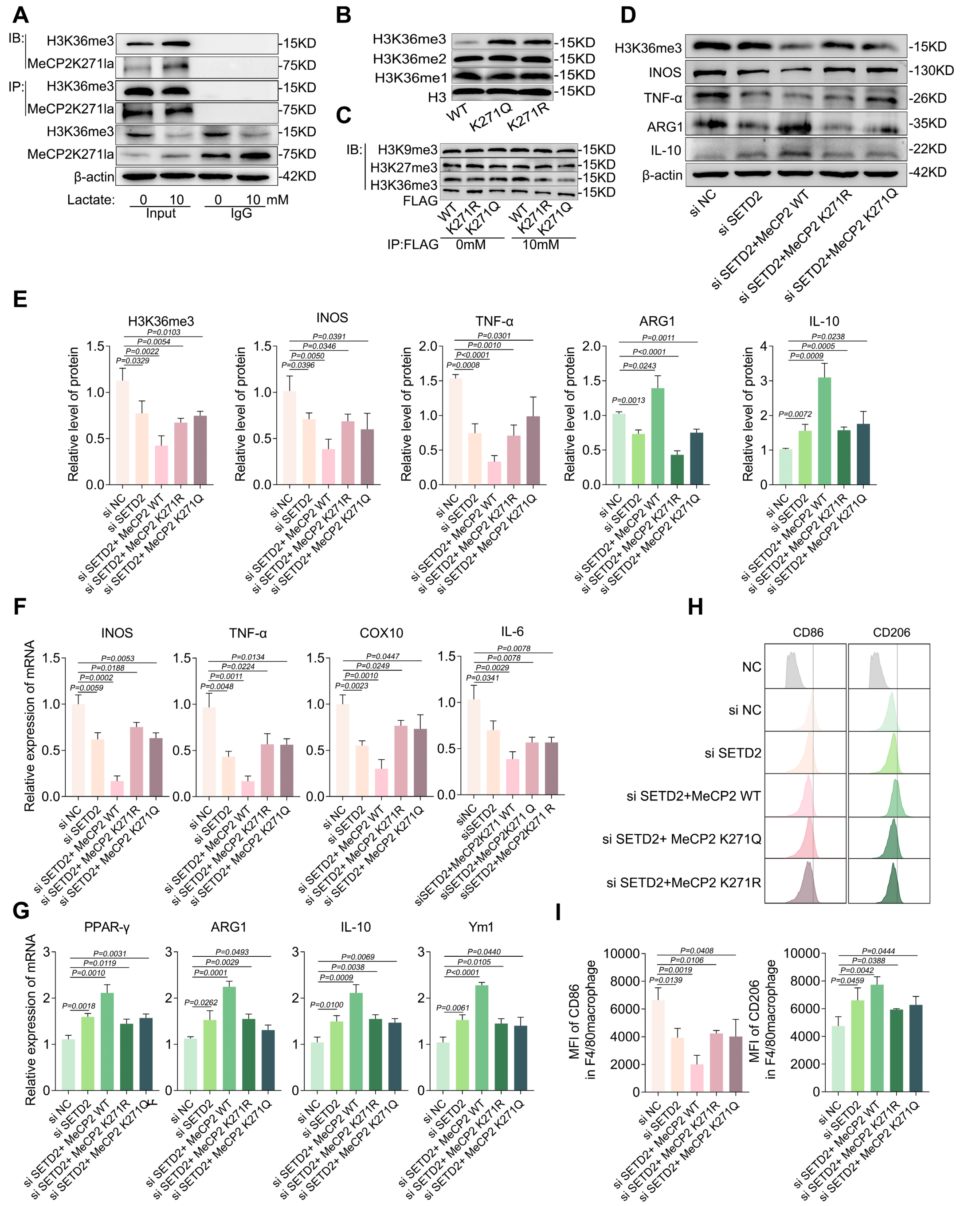

Figure S6. Lactylation of MeCP2 at K271 enhanced M2 macrophage expression in BMDMs via regulation of H3K36me3.

A. Co-IP experiment for the interaction between endogenous MeCP2 K271 lactylation and H3K36me3 in BMDMs treated with lactate. **B.** Representative levels of H3K36 methylation (me1, me2, and me3) measuring activity of ectopic expression of MeCP2 WT, K271R or K271Q plasmids. **C.** Co-IP experiment for the interaction between endogenous MeCP2 K271 lactylation and H3K36me3/H3K27me3/H3K9me3 in BMDMs transfected with the indicated plasmids and treated with or without lactate

stimulation. **D.** Western blot analysis of H3K36me3 expression in isolated PBMCs from patients with atherosclerosis treated with or without exercise training. 30 patients per group. **E, F.** Western blot analysis of RUNX1 and M1/M2 macrophage associated proteins expression **(E)** and its quantification **(F)** in BMDMs transfected with control (siNC) or P300 siRNA and treated with or without lactate for 24 h. **G.** qRT-PCR analysis of M1/M2-like genes in BMDMs MeCP2 K271WT/Q/R mutants treated with control (siNC) or SETD2 siRNA. **H, I.** Representative flow cytometric plots of M1 macrophage marker CD86 and M2 macrophage marker CD206 **(H)** and mean fluorescence intensity **(I)** in BMDMs transfected with the indicated plasmids and silenced with control (siNC) or SETD2 siRNA. All data are shown as mean±SD. Data were analyzed by one-way ANOVA with Fisher's LSD post hoc test.

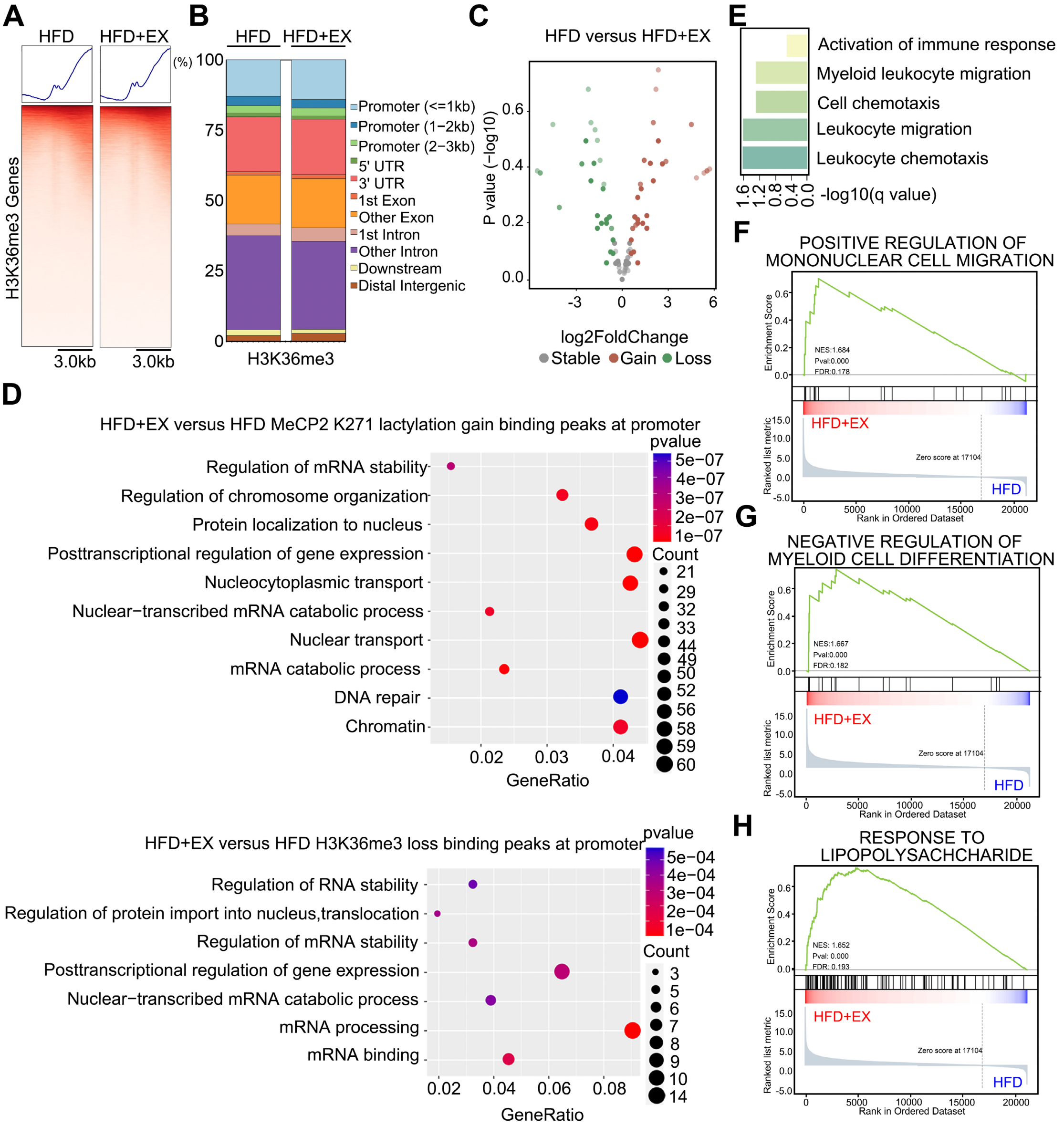

Figure S7. Lactylation of MeCP2 K271 correlated with H3K36me3 to regulate gene expression.

A. Heatmap and averaged CUT &Tag signals of H3K36me3 over±3 kb from TSS in PBMCs show the more bound transcripts of exercise trained compared to sedentary ApoE$^{-/-}$ mice. **B.** Genome-wide distribution of H3K36me3 binding peaks in ApoE$^{-/-}$ mice. **C.** Volcano plotof differential binding density of H3K36me3 in PBMCs (loss, green) (gain, brown) in comparison between exercise trained and sedentary ApoE$^{-/-}$ mice. (log2 fold change>0.58 and adj.<0.05). **D.** Dot plot showed GO analysis of increased MeCP2 K271 lactylation and H3K36me3 binding peaks at candidate target genes in PBMCs from ApoE$^{-/-}$ mice. **E.** Selected top categories from GO analysis of

ATAC regions showing increased chromatin accessibility in exercise trained compared with sedentary ApoE$^{-/-}$ mice. **F-H.** GSEA of increased accessible gene regions in exercise trained compared with sedentary ApoE$^{-/-}$ mice.

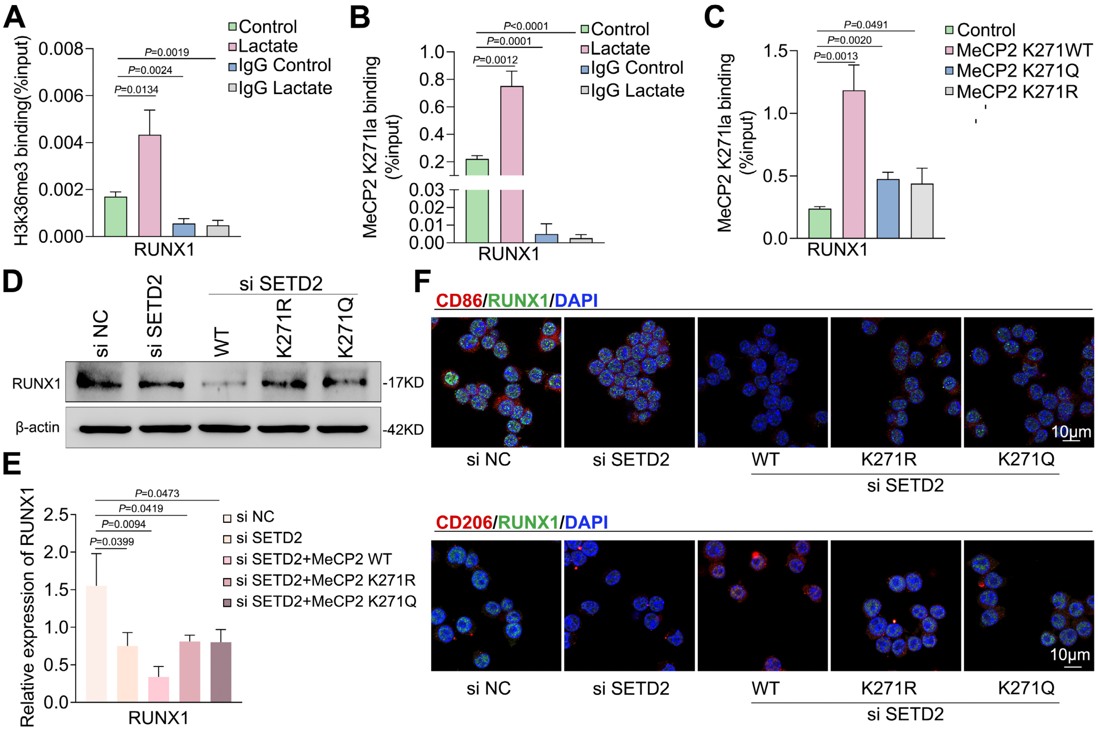

Figure S8. MeCP2 K271 lactylation correlated with H3K36me3 was important for RUNX1 regulation in BMDMs.

A, B. ChIP-qPCR of H3K36me3 **(A)**, MeCP2 K271 lactylation **(B)** and IgG, followed by qRT-PCR analysis of the promoters of RUNX1 in control and BMDMs cultured with lactate (percent input). **C.** MeCP2 K271 mutants decrease enrichments on promoters of RUNX1 in BMDMs (percent input). **D, E.** Western blot analysis of RUNX1 level in BMDMs transfected with the indicated plasmids and silenced with control (siNC) or SETD2 siRNA **(D)**, with quantification of RUNX1 **(E)**. **F.** Representative immunofluorescence co-staining images for CD86 (top) or CD206 (bottom), RUNX1 and cell nuclei in BMDMs transfected with the indicated plasmids and silenced with control (siNC) or SETD2 siRNA (D). Scale 10μm. Data were analyzed by one-way ANOVA with Fisher's LSD post hoc test.

Table S1. The list of PCR primer sequences and siRNA sequences

Primer name	Forward primer (5’→3’)	Reverse primer (5’→3’)
INOS	TCCTGGAGGTGGGCCGAAG	CCTCCACGGGCCCGGTACTC
IL-6 (mouse)	GTTCTCTGGGAAATCGTGGA	TTTCTGCAAGTGCATCATCG
COX10	CCAAGTGCTGCCGTCATTTTC	GGCTCGCAGGGATGATTTCAA
TNF-α	CCCTCACACTCAGATCATCTTCT	GCTACGACGTGGGCTACAG
Ym1	GGGCATACCTTTATCCTGAG	CCACTGAAGTCATCCATGTC
PPARγ	GGAGATCTCCAGTGATATCAGCCA	ACGGCTTCTACGGATCGAAACT
ARG1	CTCCAAGCCAAAGTCCTTAGAG	AGGAGCTGTCATTAGGGACATC
IL-10	GCTCTTACTGACTGGCATGAG	CGCAGCTCTAGGAGCATGTG
β-actin	CCATTTGCAGTGGCAAAG	CACCCCATTTGATGTTAGTG
RUNX1 (human)	CTCTGACCATCACTGTCTTCAC	CATCTAGTTTCTGCCGATGTCT
RUNX1 (mouse)	CTCTGACCATCACCGTCTTTAC	CATCTAGTTTCTGCCGATGTCT
siHDAC3(HDAC3-mus-1038)	GGAACUUCCCUAUAGUGAATT	UUCACUAUAGGGAAGUUCCTT
siP300(EP300-Mus-748)	GCCCUGGAUUAAGUUUGAUTT	AUCAAACUUAAUCCAGGGCTT
Setd2-mus-3982	GCAAGUACCAGAUUCUCUATT	UAGAGAAUCUGGUACUUGCTT
ChIP-qPCR		
RUNX1	TGAAAGCCACCAAATCCGC	AAGCAAACAGAAATCCCCTGC
ARG1	GATAGTGTAATCTGAGCAGT	GAACAATGTTCCTTATCGCTG

	TG	C
TNF-α	ATCACAGCCAAGGAAGAGC<br>C	CCAGCATGGAGCAGGGATAG
INOS	GTGGGGAAATGCTGGTCAGA	TATTCCAACACGCCCAGGAC
IL-10	GGTCAACAGGACGTGTAGCA	ACATTCGCCTAGAGTCCCCT

Table S2. Baseline Differences Between Exercise and Control Subjects of Patients.

	Exercise (30)	Control (30)	*P*
Age (y)	64.03±4.10	61.2±8.70	0.1206
Women	23.3% (7)	26.6% (8)	0.7702
Weight (kg)	71.53±11.61	71.18±12.23	0.9114
BMI (kg/m^2)	25.46±3.52	24.60±3.0	0.3234
Smokers	40% (12)	26.60% (8)	0.2812
Diabetic subjects	36.70% (11)	20% (6)	0.1574
Systolic BP (mm Hg)	138.93±16.43	135.63±20.34	0.4996
Diastolic BP (mm Hg)	86.06±11.71	82.8±13.45	0.3282
Total cholesterol (mg/dl)	4.52±1.09	4.34±1.03	0.5162
Triglycerides (mg/dl)	1.73±0.92	1.38±0.71	0.1076
HDL-C (mg/dl)	1.01±0.20	1.12±0.31	0.0954
LDL-C (mg/dl)	2.89±1.10	3.270.86	0.3712
Glucose (mg/dl)	132.58±52.31	111.63±25.04	0.0585
NT-proBNP (ng/L)	156(59, 774.5)	146(77.25, 308.5)	0.0820
LVEF (%)	61.47±7.01	61.28±3.57	0.0894
Clinical score, angiographic evaluation			
1-vessel disease	33.3% (10)	23.3% (7)	0.2674
2-vessel disease	30% (9)	26.7% (8)	0.5670
3-vessel disease	36.6% (11)	50% (15)	0.3054

Medical treatment			
ACE inhibitor	26.7% (8)	36.7% (11)	0.4136
β-blocker	50% (15)	60% (18)	0.4448
Statins	90% (27)	86% (26)	0.6936
